# Magma convection favors ephemeral melt-rich bodies within mushy reservoirs

**DOI:** 10.1038/s41467-026-74815-1

**Published:** 2026-06-25

**Authors:** Jie Chen, Lydéric France, Jean-Arthur Olive

**Affiliations:** 1 Laboratoire de Géologie, Ecole Normale Supérieure/CNRS UMR 8538, PSL Research University, Paris, France; 2 State Key Laboratory of Submarine Geoscience, School of Oceanography, Shanghai Jiao Tong University, Shanghai, China; 3 Université de Lorraine, CNRS, CRPG, Nancy, France; 4 Institut Universitaire de France (IUF), Paris, France

**Keywords:** Geodynamics, Volcanology

## Abstract

Magma convection is a mechanism that greatly enhances heat transfer from mobilizable, crystal-poor magma bodies to the surrounding immobile, crystal-rich mush reservoir of Earth’s igneous systems. As most of these systems are geophysically shown to be mush-dominated, magma convection is often omitted from thermo-kinetic models, and its role in magma evolution and eruptibility remains underexplored. Here we present 2-D numerical thermal modelling that parameterizes magma convection through a Nusselt-number approach that describes the local enhancement of heat transfers, and examine its effects in the axial mush zone of fast-spreading mid-ocean ridges. We demonstrate that magma convection, while not affecting the overall thermal regime of the mushy reservoir, significantly reduces the lifespan of individual pockets of eruptible melt to <2 years, which is two orders of magnitude shorter than in simulations without convection. Our models also show that magma convection could promote mush reheating and unlocking, potentially participating to the geochemical homogenization of heterogeneous melts extracted from the mantle. Predicted fluctuations in the occurrence and persistence of magma bodies provide insights into their highly transient nature, enhancing our ability to interpret geophysical snapshots of magma-mush systems in oceanic settings, and in other igneous systems.

## Abstract (web summary)

This study shows that magma convection greatly shortens the lifetime of mobilizable and eruptible magma at fast-spreading ridges to <2 years, highlighting the highly transient nature of magma bodies.

## Introduction

Convection in magma bodies is a fundamental process governing the thermal and chemical evolution of Earth’s igneous systems, such as oceanic spreading ridges, hotspots, continental rifts, and subduction zones, and has been a subject of scientific interest since the 1960s^1–5^. Convection can efficiently extract heat when the igneous medium is crystal-poor (i.e., magma), whereas in crystal-rich mushes approaching rheological locking, convective heat transfer is strongly suppressed and cooling primarily occurs by conduction. As a newly emplaced magma body evolves from a mobilizable and eruptible state to a locked mush reservoir, and eventually crystallizes to form a pluton^6,7^; magma convection enhances heat transfer within the magma body, exerting a key control on cooling, crystallization, and eruptibility.

Broad geophysical observations have demonstrated that most magma plumbing systems are dominated by crystal-rich mush zones^8–11^. Notable examples include fast and intermediate spreading ridges^12–14^, hotspot systems such as Hawaii^15^ and La Réunion^16^, and volcanic arcs such as Cascadia^17^ and the Andes^18^. Besides, it is common to find multiple partially-molten (i.e., crystal-poor) sills sparsely distributed across the mush zone, e.g., axial magma lenses <100 m thick at magmatically-robust, fast-spreading ridges^19–23^. These observations suggest that magma convection may be limited to isolated melt-rich sills and only exert a localized thermal impact on magma plumbing systems that commonly extend over lateral scales exceeding ~10 km.

Some key studies have explored the role of magma convection in the 1990s^2–4,24–28^. In the 21st century, thermo-kinetic modelling has played a central role in advancing our understanding of magma convection. A few studies have focused on subaerial magmatic systems, such as Krafla volcano in Iceland^29^, Andean plutons^30^, and arc volcanos^31^. At Krafla volcano in particular, magma convection within a shallow (~ 2 km) melt intrusion can substantially enhance heat transfer to the surrounding cold host rocks over timescales of 35–300 years^29^. At submarine magmatic systems, magma convection has been widely omitted in most thermo-kinetic models^32–38^. Only a few models have incorporated magma convection, either focusing on its interaction with the overlying hydrothermal circulation^39–42^, or assuming it operates throughout the entire lower crust of fast-spreading ridges^4^, a simplification that is not fully consistent with current geophysical observations^20,21,23,43^. Consequently, the broader role of magma convection within mush reservoirs in the thermal regime, crystallization, and the lifespan of eruptible magma bodies remains an open question that warrants further exploration. This issue is especially relevant to the paradigm shift from large crystal-poor magma bodies to widespread crystal-rich mush reservoirs^8,14,44,45^, as well as the recognition that newly-injected, nearly crystal-free melts likely evolve through a magma-dominated convective stage^29,46^.

**Fig. 1:**
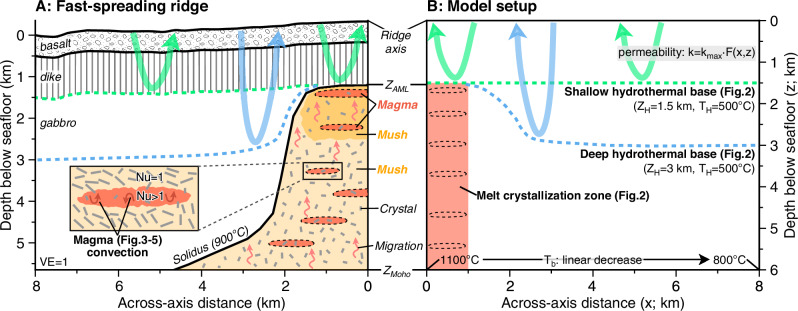
Schematic cross-section of (half of) a fast-spreading mid-ocean ridge axis. **A** Crustal structure, thermal regime, magmatic system, and potential hydrothermal system at the East Pacific Rise (EPR) 9°N (VE = 1), based on seismic tomography, reflection imaging, and magnetic data^12,13,61^. Two patterns of hydrothermal circulation are illustrated: shallow (*Z*_*H*_ = 1.5 km) in green and deep (*Z*_*H*_ = 3 km) in blue. Dark orange (1.5−2.5 km depth) and light yellow (> 2.5 km depth) zones deeper than the basaltic solidus (900°C) represent greatly (> 2 km/s) and slightly (< 1 km/s) reduced seismic P-wave velocity zones^13^, respectively. Inset sketch shows magma convection within a sill-like magma body, emphasizing the interactions between melt and crystals. **B** Cartoon of the thermal model setup. Melt sills are randomly crystallized in the melt crystallization zone between depths of the topmost seismically-imaged AML (1.5 km) and the Moho (6 km). AML axial melt lens. *Z*_*H*_ and *T*_*H*_: the maximum potential depth and temperature of the hydrothermal system. *T*_*b*_: bottom boundary temperature. *k*_*max*_: axial maximum permeability. *Nu*: Nusselt number.

In this paper, we use a 2-D numerical thermal model, *multiporo-magma*^32–34,47,48^, applied to a well-documented magmatic setting, the fast-spreading East Pacific Rise (EPR) at 9 °50’N. Its crustal structure and magma-mush system are particularly well-constrained by geophysical and geochemical data (Fig. 1A), with three volcanic eruptions documented every ~20 years, in 1991–1992, 2005–2006, and most recently in 2025^49–51^. At fast-spreading ridges, melt may crystallize throughout the lower crust^20,21,52^. The majority (~ 75%) of crystallization may occur within the upper-gabbroic layer (around 1.5–2.5 km below the seafloor), indicated by melt inclusions from EPR lavas^53^. Such a shallowly-biased melt crystallization pattern is potentially controlled by compaction^54^ and reactive melt percolation^55,56^, in which low-density melt can separate from the higher-density crystal matrix, migrate upward reactively, and crystallize at shallower depths, rather than undergoing only fractional crystallization during ascent^57^. At the EPR 9°50’N, the upper-gabbroic layer likely corresponds to a greatly reduced seismic P-wave velocity (> 2 km/s)^13^, potentially comprising multiple seismically-imaged melt sills^20,21^. In contrast, the lower gabbroic layer (> 2.5 km depth) corresponds to a slightly reduced P-wave velocity (< 1 km/s) and very few melt sills^13,20,21,43^.

## Results and discussion

### Numerical thermal model

Our 2-D numerical thermal model was previously used to explore the thermal regime and depth of magma bodies at oceanic spreading centers, by coupling hydrothermal circulation in the uppermost crust with instantaneous melt emplacement events^32–34,48^. In this paper, we incorporate an additional process, i.e., magma convection within magma bodies, parameterized using a Nusselt-number approach.

**Fig. 2:**
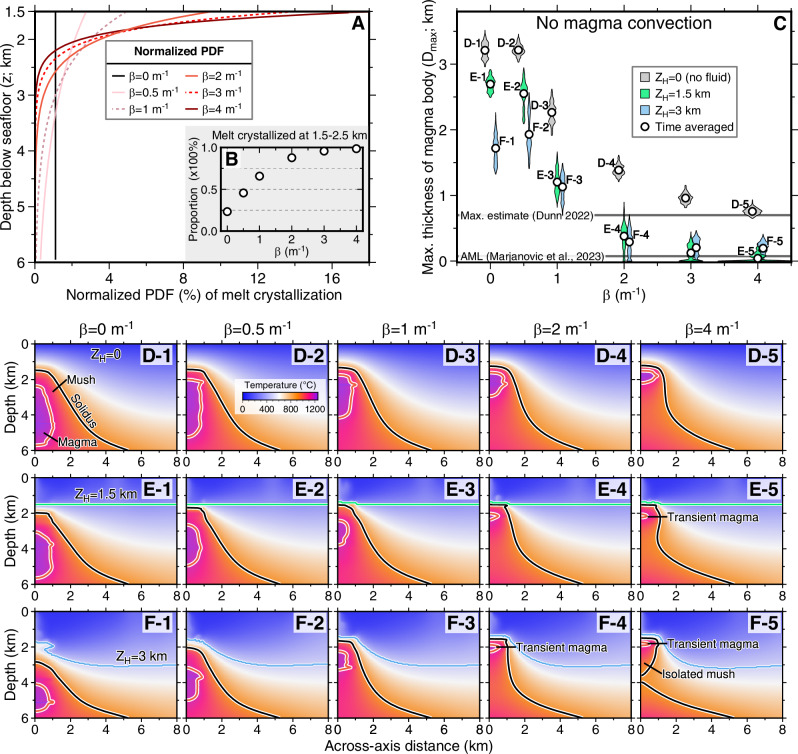
Depth of melt crystallization and numerical simulations with no magma convection. **A** Normalized probability density function (PDF) of the depth distribution of melt crystallization, based on Eq. (1). *β* = 0 corresponds to a uniform distribution, and larger *β* values indicate a bias towards shallower melt crystallization. **B** Total proportion of melt crystallized at 1.5–2.5 km vs. *β*. **C** Violin plot for maximum thickness (*D*_*max*_) of the magma body vs. *β*, with three hydrothermal configurations: *Z*_*H*_ = 0 (no fluid), 1.5, and 3 km. Open circles represent time-averaged *D*_*max*_ over 30 kyrs. The ideal range of *D*_*max*_ between two horizontal lines is constrained by seismic tomography and reflection observations^13,43^. **D**–**F** Model snapshots of quasi-steady-state thermal regimes at *Z*_*H*_ = 0 (**D**), 1.5 km (**E**), and 3 km (**F**), with *β* increasing from 0 to 4 m^−1^. Red lines outline the magma body (*X* < *X*_*c*_). Black lines mark the solidus isotherm (900 °C). The mush zone is in between. Green (**E**) and blue (**F**) lines represent the base of the hydrothermal system at *Z*_*H*_ = 1.5 and 3 km, respectively. Transient magma bodies are indicated.

In our simulations, which represent half of a vertical section across the ridge axis, horizontal melt sills, 1000 m in width (*W*_*sill*_) and 50 m in thickness (*H*_*sill*_), are emplaced every 200 years at random depths (*z*) within a 1-km-wide axial region (Fig. 1B). This corresponds to a time-averaged input of material of 250 m^2^/kyr for the half plate at EPR 9 °50’N, enough to build a 4.5 km-thick lower crust (“Methods”). Melt sills are emplaced at depths between the topmost seismically-imaged axial melt lens (AML; *Z*_*AML*_ = ~ 1.5 km) and the Moho (*Z*_*Moho*_ = ~6 km)^13,19,21^. The depth distribution of melt sills (or of melt crystallization) is prescribed by a normalized probability density function (PDF) that decays exponentially with depth:

1 $${PDF} \sim {e}^{-\beta ({\rm{z}}-{{\rm{z}}}_{{AML}})},{\rm{z}}\in [{{\rm{z}}}_{{AML}},{{\rm{z}}}_{{Moho}}]$$

where *β* is the decay rate (in m^−1^). This parameter determines the time-averaged proportion of melt crystallized at a given depth (Fig. 2A) or within a given depth range (Fig. 2B). *β* = 0 corresponds to a uniform depth distribution of melt crystallization, and larger *β* values represent a more pronounced bias towards shallower melt crystallization (Fig. 2A). This leads to a total proportion of melt crystallized at 1.5–2.5 km (i.e., the upper-gabbroic layer), increasing from 23 to 98%, as *β* increases from 0 to 4 m^−1^ (Fig. 2B).

**Fig. 3:**
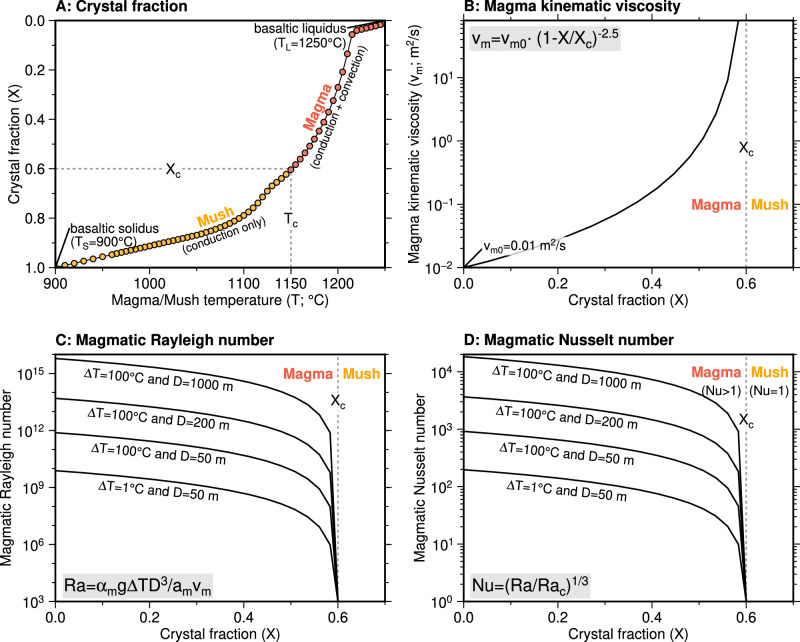
Relationships among temperature, crystal fraction, magma viscosity, Rayleigh number, and Nusselt number. **A** Temperature (*T*) vs. crystal fraction (*X*), spanning from the basaltic solidus (*T*_*S*_ = 900 °C) to the liquidus (*T*_*L*_ = 1250 °C)^58^. Magma and mush are defined by *X* < *X*_*c*_ (0.6) and *X* > *X*_*c*_, respectively. **B**–**D** Crystal fraction vs. magma kinematic viscosity (${\nu }_{m}$), Rayleigh number (*Ra*), and Nusselt number (*Nu*), calculated using Eqs. (2)–(4). Solid lines in (**C**) and (**D**) show examples of analytical results for ∆*T* = 1−100 °C and *D* = 50–1000 m. See Table S1 for parameter notations and values.

Crystal fraction (*X*) of magma and mush is prescribed based on constraints from a thermodynamic model and evolves with temperature (*T*) according to a nonlinear *T*-*X* relationship^58^ (Fig. 3A), in which *X* increases from 0 at the basaltic liquidus (*T*_*L*_) of 1250 °C to 1 at the basaltic solidus (*T*_*S*_) of 900 °C (“Methods”). We assume that a magma body is mobilizable below a critical crystal fraction value (*X*_*c*_) of 0.6^59,60^, corresponding to a critical temperature (*T*_*c*_) of 1150 °C (Fig. 3A). By contrast, the immobile mush zone has a high crystal fraction, corresponding to *X* > *X*_*c*_ and *T* < *T*_*c*_ (Fig. 3A).

Hydrothermal circulation is confined within a shallow permeable layer, obeying Darcy’s law (“Methods”). Permeability is greatest (*k*_*max*_ = 10^−14^ m^2^) at the seafloor above the axis (*x* = 0), and decays exponentially with both depth and across-axis distance (Fig. S1; “Methods”). Hydrothermal fluids are restricted to a maximum depth (*Z*_*H*_), and fluid circulation cannot occur above a maximum hydrothermal temperature (*T*_*H*_) of 500 °C based on constraints from magnetic polarity boundaries observed during gabbro cooling^61^.

### Models without magma convection

We first run numerical models with no magma convection. We tested 6 values of *β* (depth distribution of magma input) at 0, 0.5, 1, 2, 3, and 4 m^−1^, and 3 values of *Z*_*H*_ (hydrothermal layer thickness) at 0 (no hydrothermal convection), 1.5, and 3 km, amounting to 18 test cases (Fig. 2). We measure the maximum thickness (*D*_*max*_) of the magma body, i.e., the region where *X* < *X*_*c*_, every 0.1 year over a 30 kyrs period following the establishment of a quasi-steady thermal regime (e.g., stabilized mush geometry and hydrothermal circulation). This time window was empirically found sufficient to capture the full range of temporal variations in the magma body.

We present the results using violin kernel density plots alongside time-averaged values (Fig. 2C). For *β* = 0, a persistent, long-lived magma body persists near the model bottom (i.e., Moho) regardless of the hydrothermal system configuration (Fig. 2D-1–F-1). As *Z*_*H*_ increases from 0 (no hydrothermal flow) to 1.5 and 3 km, the efficiency of hydrothermal cooling increases, resulting in a colder thermal regime and a reduction in *D*_*max*_ from >3 to ~1.8 km (Fig. 2C). However, such a large, long-lived magma body contradicts seismic observations that suggest a mush-dominated lower crust at the EPR 9°N^13^. Besides, the axial solidus, in simulations with *Z*_*H*_ = 1.5 and 3 km (Fig. 2E-1, F-1), lies deeper than the topmost seismically-imaged AML, suggesting that the predicted thermal regime in the upper-gabbroic layer is too cold.

**Fig. 4:**
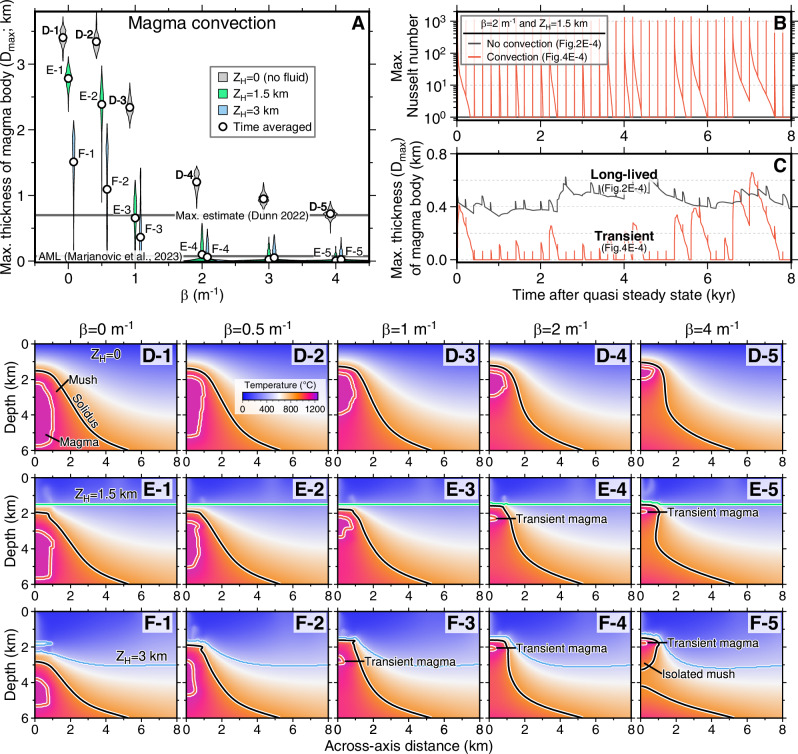
Numerical simulations incorporating magma convection. The configurations of melt crystallization distribution and hydrothermal circulation are identical to those in the models with no magma convection (Fig. 2). **A** Violin plot for maximum thickness (*D*_*max*_) of the magma body vs. *β*. **B**, **C** Temporal evolution of maximum Nusselt number and *D*_*max*_ after quasi steady state over 8 kyrs, comparing models with (red lines) and without (black lines) magma convection. **D**–**F** Model snapshots of quasi-steady-state thermal regimes at *Z*_*H*_ = 0 (**D**), 1.5 km (**E**), and 3 km (**F**), with *β* increasing from 0 to 4 m^−1^. Red contour outlines the magma body, defined as regions with crystal fraction (*X*) < 0.6, where magma convection is parameterized (*Nu* > 1).

**Fig. 5:**
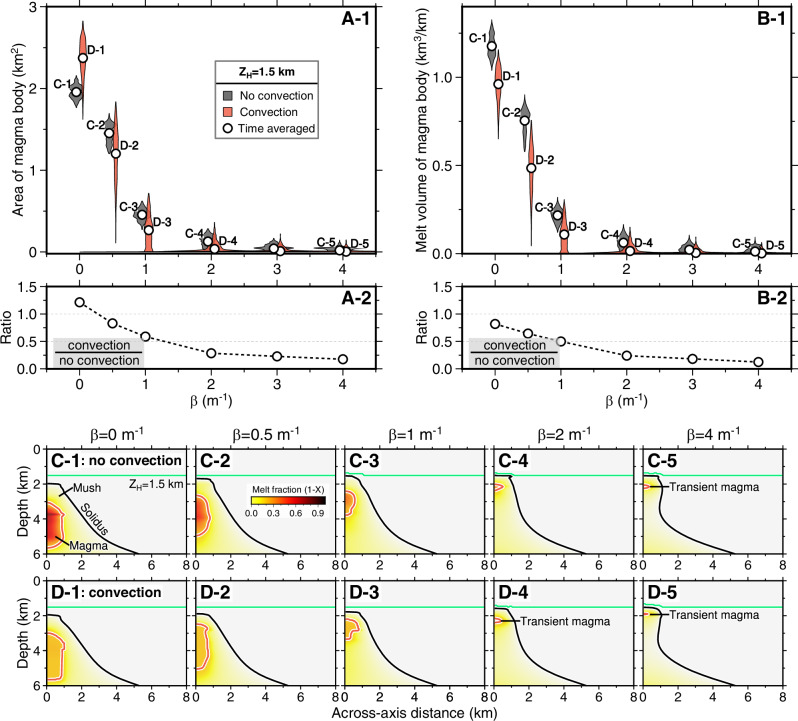
Comparison of simulations with and without magma convection at *Z*_*H*_ = 1.5 km. **A** Violin plot for the area of the magma body vs. *β*, as well as the ratio of its time-averaged area (over 30 kyrs) with magma convection to no convection. **B** Violin plot and ratio for the melt volume of the magma body. **C**, **D** Model snapshots of quasi-steady-state melt fraction (1–*X*), without (**C**) and with (**D**) magma convection, corresponding to temperature snapshots in Figs. 2E and 4E, respectively. See simulations at *Z*_*H*_ = 0 and 3 km in Figs. S8 and S9. Green lines represent the base of the hydrothermal system at *Z*_*H*_ = 1.5 km.

Simulations with increasing *β* show that the magma body becomes thinner and shallower, for a given hydrothermal configuration (Fig. 2). For example, at *Z*_*H*_ = 1.5 km (Fig. 2E), the thermal regime in the lower gabbroic layer (> 2.5 km depth) becomes colder with increasing *β*, while the upper-gabbroic layer becomes hotter. At *β* = 0–1 m^−1^, the magma body remains persistent, with a thickness *D*_*max*_ and a depth exceeding 1 and 2 km, respectively (Fig. 2E-1–3). In contrast, at *β* = 2–4 m^−1^, the magma body is mostly confined to depths shallower than 2 km, with a *D*_*max*_ reduced to <0.5 km (Fig. 2E-4, 5). These thin and shallow magma bodies fall within the range constrained by seismic tomography and reflection^13,43^ (Fig. 2C). Notably, at *β* = 4 m^−1^, the magma body is transient and occasionally disappears (Fig. 2E-5). Simulations with *Z*_*H*_ = 3 km exhibit overall colder thermal regimes compared to those with *Z*_*H*_ = 1.5 km, due to more efficient heat extraction by a deeper-penetrating hydrothermal system (Fig. 2F). Specifically, at *β* = 0 m^−1^ (*Z*_*H*_ = 3 km), the mush zone and the magma body become deeper and smaller, and at *β* = 4 m^−1^ (*Z*_*H*_ = 3 km), an isolated mush zone forms within the upper-gabbroic layer. In simulations with no hydrothermal fluid (*Z*_*H*_ = 0; Fig. 2D), the thermal regime is overall hotter, and the magma body remains persistent and thicker, compared to those with *Z*_*H*_ = 1.5 and 3 km (Fig. 2E, F).

### Magma convection with Nusselt parameterization

We parameterize the enhancement of heat transfer due to magma convection using a conductive enhancement factor—the magmatic Nusselt number (*Nu*)—which represents the ratio of total (conductive plus convective) to conductive-only heat transfer within the mobilizable magma body^41,42^. The heat transfer in the magma body is conductive and convective (*Nu* > 1), while in the mush zone, it is only conductive (*Nu* = 1).

The Nusselt number for a lateral magma body in a 2-D cross section (e.g., this study) can be calculated using an analytical solution^41,42^, mainly depending on magma kinematic viscosity (${\nu }_{m}$ in m^2^/s), the temperature difference between the magma body and the surrounding mush (∆*T*), and the thickness of the magma body (*D*). In numerical simulations, ∆*T* is measured between *T*_*c*_ and the magma body’s maximum temperature, with *D* representing its average thickness.

Magma kinematic viscosity (${\nu }_{m}$) relates to the crystal fraction (*X*; Fig. 3B), expressed as^59,60,62^:

2 $${\nu }_{m}={\nu }_{m0}{\left(1-\frac{X}{{X}_{c}}\right)}^{-2.5},$$

where ${\nu }_{m0}$ is ${\nu }_{m}$ of liquid basaltic magma (*T* = *T*_*L*_ and *X* = 0) at 0.01 m^2^/s^63–65^. ${\nu }_{m}$ in the magma body increases with *X*, and approaches infinity as *X* approaches *X*_*c*_ (Fig. 3B). In the model, the 2-D ${\nu }_{m}$ field evolves with *X*. The 2-D fields of Rayleigh (*Ra*) and Nusselt (*Nu*) numbers within the magma body (Fig. 3C, D) then can be calculated as:

3 $$\it {Ra}=\frac{{\alpha }_{m}g\Delta T{D}^{3}}{{a}_{m}{\nu }_{m}},$$

4 $$\it {Nu}={\left({Ra}/{{Ra}}_{c}\right)}^{1/3},$$

where ${\alpha }_{m}$ is the thermal expansion coefficient, *g* is the gravity acceleration, ${a}_{m}$ is the thermal diffusivity, and *Ra*_*c*_ is the critical value of *Ra* at 10^3^. Figure 3C, D illustrate the relationship between *Ra*, *Nu*, ∆*T* (between 1 and 100 °C), and *D* (between 50 and 1000 m). For example, at ∆*T* = 100 °C and *D* = 200 m, *Ra* decreases from >10^13^ at *X* = 0 to 10^3^ at *X* = *X*_*c*_, accompanied by a decrease in *Nu* from >3 × 10^3^ to 1. This demonstrates that a Nusselt-based parameterization of heat extraction dynamically captures the transition from convection-dominated to conduction-dominated heat transfer as magma cools and crystallizes. We note that *Ra* in our model only accounts for thermally induced convection, whereas compositional convection arising from density contrasts between basaltic melts and crystals may generate larger *Ra*^29^.

We run models incorporating magma convection (Figs. 4 and 5), using *Nu* to parameterize the enhancement of conductive heat transfer (*Nu* > 1 within the crystal-poor magma body and *Nu* = 1 elsewhere), as implemented in Eq. (6) (see “Methods”). The *Nu* field evolves over time in response to magma cooling, crystallization, and successive melt emplacement. All models use the same configurations of melt crystallization distribution and hydrothermal circulation as those without magma convection, resulting in the same set of 18 test cases (Fig. 2). To first order, their overall thermal regimes are very similar (Figs. 2D, E and 4D, E). This confirms that a shallowly-biased melt crystallization pattern (e.g., *β* = 2 m^−1^ in Fig. 4E-4, F-4) is necessary to reproduce the magma plumbing architecture of the EPR 9 °N, as constrained by seismic observations^13,20,21,43^. This pattern is also consistent with geochemical signatures from fast-spreading ridges^53,66,67^, suggesting that most crystallization and more efficient melt mixing occur in the upper-gabbroic layer (dark orange zone in Fig. 1A) compared to deeper levels.

We also measure the maximum thickness (*D*_*max*_), cross-sectional area, and melt volume of the magma body, all of which exhibit greater variability in magma-convection simulations, and this variability increases as *Z*_*H*_ increases (Figs. 2C, 4A, 5, S8, and S9). Again, taking the simulations with *Z*_*H*_ = 1.5 km as an example (Figs. 4E and 5), magma convection is vigorous at *β* = 0–0.5 m^-1^, with a thick magma body of >2 km and a high magmatic Nusselt number between 20 and >10^4^ (Fig. S3E-1, 2), allowing efficient heat extraction from the magma to the surrounding mush. This increases the time-averaged area of the magma body, but reduces its total melt volume (i.e., the magma body has a lower temperature and a greater crystal fraction), compared to simulations with no magma convection (Figs. 5A, B and S6). At *β* = 2–4 m^−1^, the magma body in magma-convection simulations becomes more transient with lower and more variable Nusselt numbers between 1 and 10^3^ (Fig. 4B, C), and its time-averaged area and melt volume are all reduced by >50% (Fig. 5A, B). In simulations with *Z*_*H*_ = 3 km, magma bodies can be transient at a lower *β* (≥1 m^−1^) than in those with *Z*_*H*_ = 1.5 km (Fig. 4D vs Fig. 4E). In simulations with no hydrothermal circulation (*Z*_*H*_ = 0), the magma bodies are persistent at any *β* values, and magma convection always increases their areas and reduces their melt volumes (Fig. S8).

In summary, for a given *β* (i.e., the depth distribution of melt crystallization, in m^−1^), simulations incorporating magma convection produce a more transient magma body system than those without convection, with deeper hydrothermal cooling further enhancing the transience. This pronounced transience, such as the maximum thickness of magma body at the simulation with *β* = 2 m^−1^ and *Z*_H_ = 1.5 km varying between 0 and 0.6 km over 8 kyr (Fig. 4C), is also consistent with petro-structural observations from fossilized oceanic crust^67^. By contrast, the geometry of the mush reservoir is weakly influenced by magma convection (e.g., Figs. 2 and 4), with the axial profiles of temperature and crystal fraction within the mush zone largely similar in configurations with and without magma convection (Figs. S5–S7). Instead, it is primarily controlled by the *β* value and the hydrothermal configuration; a higher *β* value and a deeper hydrothermal system, i.e., a closer spatial proximity between melt emplacement and the hydrothermal system, lead to more efficient hydrothermal heat extraction^33^ and, consequently, to a smaller mush zone (Fig. 4D–F).

### Implications for the lifespan of eruptible magma bodies

**Fig. 6:**
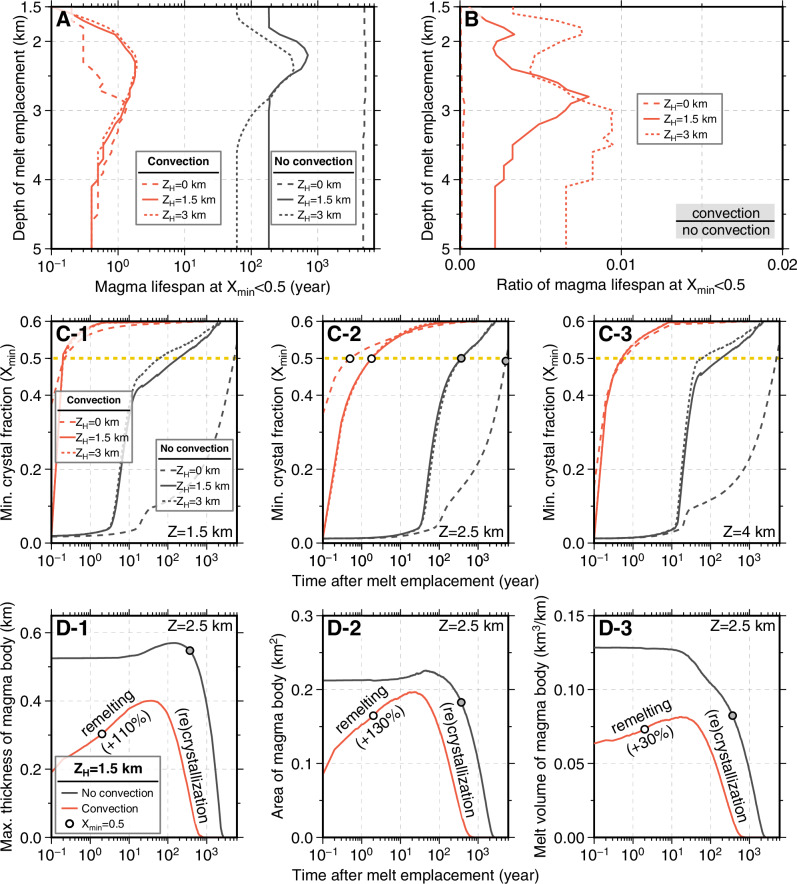
Magma lifespan with and without magma convection at *β* = 2 m^−1^. **A** Magma lifespan at depths of 1.5–5 km, defined as the duration during which the minimum crystal fraction (*X*_*min*_) remains between 0 and 0.5. See legend for configurations of magma convection and hydrothermal circulation. **B** Ratio of magma lifespan with convection to no convection. **C** Temporal evolution of *X*_*min*_ after melt emplacement at depths of 1.5, 2.5, and 4 km. Dashed yellow lines correspond to the threshold of magma lifespan at *X*_*min*_ = 0.5. **D** Temporal evolution of the maximum thickness, area, and melt volume of the magma body (defined as the region where *X* < 0.6), for melt emplacement at 2.5 km and *Z*_*H*_ = 1.5 km. Open and gray circles mark the time when *X*_*min*_ reaching 0.5 at *Z* = 2.5 km for convection and no convection models, respectively.

The lifespan of a mobilizable magma body defines the time window during which the magma remains eruptible or extractable following a new melt emplacement event^6,7^, and has strong implications in terms of risk mitigation in volcanically active provinces. To investigate the impact of convection on magma lifespan, we run additional simulations involving Monte–Carlo analysis of single-injection events, each initialized from the final state of the previous simulations. Specifically, we randomly select 50 quasi-steady-state temperature fields from both no-magma-convection (Fig. 2D-4–F-4) and magma-convection simulations (Fig. 4D-4–F-4), which are conducted at *β* = 2 m^−1^ (the value that best reproduces seismological observations of the EPR 9°N) and *Z*_*H*_ = 0, 1.5, and 3 km. For each selected temperature field, we run 36 simulations, each with a single injection of a 1000-m-wide and 50-m-thick melt sill emplaced at a depth between 1.5 and 5 km, with 0.1 km increments of depth. Each new simulation is then run forward in time until the newly emplaced magma body entirely transitions to a mush (i.e., *X* > 0.6). We define magma lifespan as the time span during which the minimum crystal fraction (*X*_*min*_) remains between 0 and 0.5, and calculate the average magma lifespan across the 50 randomly selected temperature fields (Fig. 6A). Additional magma lifespan estimates using thresholds of *X*_*min*_ at 0–0.4 and 0–0.55 are also provided for comparison (Fig. S10). We also track the temporal evolution of *X*_*min*_, maximum thickness (*D*_*max*_), and area and melt volume of the magma body throughout the model runtime, all of which are averaged across the 50 Monte–Carlo simulations (Fig. 6C, D).

In simulations with no magma convection and *Z*_*H*_ = 1.5 km (black solid line in Fig. 6A), magma lifespan increases from 180 to 700 years as the depth of melt emplacement increases from 1.5 to 2.2 km depth, then decreases to 180 year at 3.5 km depth, and finally remains relatively constant for emplacement depths between 3.5 and 5 km. In contrast, simulations with magma convection show magma lifespans only ranging from 0.1 to 2 years with a peak at 2.5 km (red solid line in Fig. 6A), and significantly accelerated crystallization at all depths (Fig. 6C). As a result, magma convection reduces magma lifespan by about 2 orders of magnitude everywhere (Fig. 6B), which persists in simulations with deeper hydrothermal circulation (*Z*_*H*_ = 3 km with dotted lines in Fig. 6). For simulations with no hydrothermal circulation (*Z*_*H*_ = 0 with dashed lines in Fig. 6), magma lifespan with no magma convection can reach up to 5000 years, and is maintained by the persistence of a large, pre-existing magma body (Fig. 2D-4). However, when convection is incorporated, the magma lifespan is significantly reduced to only ~1 year (Fig. 6A), particularly at depths of 1.5–3 km where the pre-existing magma body is located (Fig. 4D-4). We expect that compositional convection between magma and crystals would further decrease the lifespan of crystal-poor magma domains^29^; therefore, the magma lifespan obtained in our model, which does not include this additional process, should be regarded as a maximum estimate. In other words, the transience of eruptible magma bodies in nature may be even greater than estimated in our model.

Our convection models are, therefore, able to predict individual horizontal melt pockets with a lifespan less than 2 years under the context of the fast-spreading EPR 9 °N (Figs. 6A and S10), which is two orders of magnitude shorter than in no-convection models. Our results can also be directly linked to the timing of observed volcanic eruptions at the EPR 9 °N, which occur every ~20 years (1991–1992, 2005–2006, and most recently in 2025)^49–51^. These results imply that continuous magma recharge, or very frequent recharge events are required in between those various eruptive stages. Notably, in the absence of hydrothermal circulation (*Z*_*H*_ = 0 km), the reduction of magma lifespan due to convection is even more pronounced (Fig. 6B), suggesting that our findings for oceanic spreading centers may be applicable to non-oceanic settings where hydrothermal activity is limited^29,68^. In particular, our conclusions on the lifespan of melt-rich bodies and their transience can potentially be extended to other mafic systems, with or without hydrothermal systems, where episodic magma recharges through mushy systems are identified. It may be the case at slow spreading oceanic centers^69,70^, ocean-continent transitions^68,71,72^, or other continental settings^8^. This insight, in turn, enhances our understanding of volcanic eruptibility and improves the interpretation of geophysical snapshots of magma plumbing architectures. In all simulations with magma convection (Fig. 6A), the fraction of time during which a given portion of the igneous reservoir remains eruptible varies from 0.05 to 1%, which we estimated by dividing the magma lifespan by the melt emplacement interval of 200 years.

Additionally, a newly-emplaced melt body can act as a heat source, remelting the surrounding crystal mush by magma convection and rapid crystallization over a short timescale (Fig. 6C). This remelting stage participates to the local unlocking of the crystal-mushes, rejuvenating it, and eventually increasing the *D*_*max*_ and area of the eruptible magma by up to 110 and 130%, respectively, over ~50 years in simulations with melt crystallized at 2.5 km and *Z*_*H*_ = 1.5 km (Fig. 6D). At the same time, the melt volume in the magma body increases by 30% (Fig. 6D). In contrast, these properties are relatively stable during this period in simulations with no magma convection. The resulting magma body, including remelted materials, then undergoes slow (re)crystallization by nearly conductive cooling, over a longer timescale (~ 600 years; Fig. 6D). This suggests that some crystals, especially at the upper-gabbroic layer with a high *β* value, may experience multiple episodes of remelting and recrystallization over a time span of several years, thereby enhancing melt mixing and homogenizing geochemical signatures. This could potentially explain some of the textural resorption or assimilation features that are observed in fossilized plutonic sections of fast-spreading ridges^66,73^, and participate to the progressive isotopic homogenization of mantle magmas upon ascent through the igneous reservoir^74^.

Our numerical thermal models highlight that considering magma convection in crystal-poor (i.e., melt-rich) magma bodies is essential to track the spatial, thermal, and temporal dynamics between magma and mush. Magma convection not only significantly shortens the lifespan of eruptible magma bodies by two orders of magnitude (< 2 years), but also triggers local remelting and unlocking of the surrounding mush reservoir. Together with a shallowly-biased melt crystallization pattern (i.e., high *β* values) at fast-spreading ridges, magma convection could increase the melt volume of the eruptible magma body by up to 30% and promotes melt mixing and geochemical homogenization over the upper-gabbroic layer. We therefore propose that magma convection is an inherent process in oceanic magma-mush systems, and likely in other geodynamic contexts as well. The highly transient nature of magma bodies appears to be a universal characteristic, providing insights into volcanic eruptibility and suggesting a potential link between geophysical snapshots and the broader architecture of magma plumbing systems across different geodynamic contexts.

## Methods

### Model setup

Our 2-D numerical approach, *multiporo-magma*, is an extension of the previously developed model, *multiporo*^32–34,47,48^, incorporating additional processes related to magmatic dynamics. The numerical domain represents one half of a vertical section across the fast-spreading East Pacific Rise (EPR) 9°N axis. It is 6 km tall and 8 km wide, and discretized into 50-m wide cells (see Fig. 1B for model setup and Table S1 for a summary of parameter notations and values). The top boundary (i.e., seafloor) temperature (*T*_*0*_) is fixed to 0 °C, and the bottom boundary temperature (*T*_*b*_) is imposed at 1100 °C at the ridge axis and decreases linearly to 800 °C toward the right boundary (Fig. 1B). The initial temperature field increases linearly from *T*_*0*_ to *T*_*b*_, except within the melt crystallization zone where it is set to 1100 °C uniformly (Fig. 1B). Dike intrusions and off-axis melt emplacement are neglected in the model, as they mainly affect the thermal regime outside the main magma-mush system^32^. Heat transfer can occur by conduction throughout the entire numerical domain, advection by hydrothermal circulation in the uppermost crust (see section below), and advection modeled as enhanced diffusion within the mobilizable magma body (Fig. 3). All simulations are run until they reach quasi-steady-state thermal regimes.

To simulate hydrothermal circulation, we apply a finite volume method to solve for conservation of mass and energy in fluid-saturated, non-deforming porous medium obeying Darcy’s law:

5 $${\boldsymbol{\nabla }}\cdot \left(-\frac{k}{{\mu }_{f}}\left({\boldsymbol{\nabla }}{\boldsymbol{P}}-{\rho }_{f}{\boldsymbol{g}}\right)\right)=0,$$

where *k* is the permeability, *μ*_*f*_ is the viscosity of the fluids, and *ρ*_*f*_ is the density of the fluids. The gradient of pressure (${\boldsymbol{\nabla }}$**P**) and the acceleration of gravity (***g***) in bold are vectors. The fluid velocity (***v***_***f***_) corresponds to the term inside the divergence operator. The thermodynamic properties of the hydrothermal fluids, including *ρ*_*f*_, correspond to those of a 3.2 wt% NaCl-H_2_O mixture^75,76^, evolving with the temperature and pressure fields of the simulation. The viscosity of the hydrothermal fluid is determined by temperature, approximated as that of pure water^77^. The top boundary (i.e., seafloor) is set to allow hydrothermal fluids to escape (i.e., open top boundary condition).

To evolve the temperature field *T*, we solve Eq. (5) with the heat equation:

6 $$\left(\left(1-\phi \right){\rho }_{s}{C}_{s}+\phi {\rho }_{f}{C}_{f}\right)\frac{\partial T}{\partial t}+{{\rho }_{f}{C}_{f}{\boldsymbol{v}}}_{{\boldsymbol{f}}}\cdot {\boldsymbol{\nabla }}{\boldsymbol{T}}={Nu}\lambda {\nabla }^{2}T+{H}_{M},$$

where *ϕ* is porosity. ${\rho }_{s}$ and ${\rho }_{f}$ are the density of solid and hydrothermal fluids, and ${C}_{s}$ and ${C}_{f}$ are the heat capacity of solid and hydrothermal fluids. *λ* is the thermal conductivity, and *Nu* is the Nusselt number, expressed in Eq. (4). *H*_*M*_ is the term for latent heat from magma crystallization, expressed in Eq. (11).

### Permeability structure

Permeability is set to exponentially decrease from *k*_*max*_ = 10^−14^ m^2^ at the axial seafloor (i.e., axial maximum permeability), horizontally and vertically. Horizontally, we assume that crustal porosity is reduced due to mineral precipitation by hydrothermal circulation, and thus the seafloor permeability (*k*_*0*_) is set to decrease from the ridge axis (*k*_*max*_) to the right boundary by one order of magnitude, following:

7 $${k}_{0}(x)={k}_{\max }{10}^{\left(-x/{x}_{b}\right)},$$

where *x* is the across-axis distance, and *x*_*b*_ is the across-axis distance of the right model boundary (i.e., 8 km). Vertically, we assume permeability is pressure (i.e., depth)-decreasing^78,79^, expressed as:

8 $$k(x,{\rm{z}})={k}_{0}\left(x\right).{\rm{\cdot }}{e}^{-{P}_{{eff}}({\rm{z}})/{P}_{d}},$$

9 $${P}_{eff}=({\rho }_{r}-{\rho }_{f}){g}{\rm{z}},$$

where *P*_*eff*_ is the effective pressure, determined by the constant rock density (*ρ*_*r*_), the fluid density of the hydrothermal system (*ρ*_*f*_), and the depth (*z*). *P*_*d*_ is the normalized pressure sensitivity coefficient, which is set to *P*_*d*_ = 30 MPa near the ridge axis (i.e., *x* = 0–1 km) and *P*_*d*_ = 10 MPa beyond. In numerical simulations, permeability evolves with temperature and pressure fields.

The maximum potential depth extent of hydrothermal circulation (*Z*_*H*_) is set to 0 (no fluid circulation), 1.5, and 3 km. The maximum temperature at which fluids can circulate (*T*_*H*_) is set to 500 °C, based on constraints from magnetic polarity boundaries observed during gabbro cooling^61^. *Z*_*H*_ and *T*_*H*_ together define the boundary between permeable and impermeable lithosphere, e.g., a fracture front, beneath which permeability is assigned a very low value (*k* = 10^−25^ m^2^, effectively impermeable) in the numerical model. Figure S1 shows snapshots of permeability structures that correspond to models in Fig. 2E (*Z*_*H*_ = 1.5 km) and Fig. 2F (*Z*_*H*_ = 3 km). The minimum permeability, located at *x* = 8 km, reaches 5.5 × 10^−17^ and 1.5 × 10^−18^ m^2^ for the two cases, respectively.

### Melt crystallization and heat input

Melt sills are instantaneously emplaced within the axial melt crystallization zone to match a lower-crustal melt flux of φ = ~250 m^2^/kyr for the half plate at the EPR 9°N (Fig. 1). Each melt is a horizontal sill, with 1000 m in width (*W*_*sill*_) and 50 m in thickness (*H*_*sill*_), corresponding to a melt emplacement interval of *τ* = 200 years, calculated by:

10 $$\tau=\frac{W_{sill}H_{sill}}{\varphi }.$$

The depth of melt crystallization for each event is randomly selected, and its distribution follows a normalized probability density function (PDF; Fig. 2A).

Each sill emplacement resets the temperature to a uniform liquidus temperature (*T*_*L*_) of 1250 °C, corresponding to a crystal fraction (*X*) of 0. This melt then cools and crystalizes in-situ, releasing specific and latent heat. The amount of latent heat (*H*_*M*_) that is released upon cooling from *T*_*L*_ to a solidus temperature (*T*_*S*_) of 900 °C is expressed in the heat conservation equation as^80^:

11 $${H}_{M}=\frac{-L{\rho }_{M}}{{T}_{L}-{T}_{S}}\frac{\partial T}{\partial t},$$

where *L* is the latent heat of crystallization, and *ρ*_*M*_ is the magma density. The latent heat, in addition to the specific heat of magma and hot rocks, fuels hydrothermal circulation.

### Lab measurements on temperature and crystal fraction of basaltic Magma and Mush

Temperature (*T*) and crystal fraction (*X*) follow a nonlinear relationship, in which *X* increases from 0 at the basaltic liquidus (*T*_*L*_) of 1250 °C to 1 at the basaltic solidus (*T*_*S*_) of 900 °C (Fig. 3A). This relationship is based on a fractional crystallization model implemented with the thermodynamic model Rhyolite-MELTS, by using a relatively primitive MORB composition^58^. We performed the modelling at redox conditions one log unit below the fayalite-magnetite-quartz oxygen buffer, at a pressure of 2 kbar, and a water content of 0.1 wt% consistent with the conditions prevailing at fast-spreading oceanic centers^58^.

## Supplementary information

Supplementary

Transparent Peer Review file

## Data Availability

The simulation videos of temperature and crystal fraction fields generated in this study have been deposited in the Figshare (10.6084/M9.FIGSHARE.30442703).

## Code availability

The source code used in this study has been deposited in the Figshare (10.6084/M9.FIGSHARE.30442703).
