# Supplementary material for: Magma convection favors ephemeral melt-rich bodies within mushy reservoirs
Source: Nat Commun. 2026 Jun 25;17:7957. doi: 10.1038/s41467-026-74815-1 (PMC13448659; doi:10.1038/s41467-026-74815-1)
Supplement: Supplementary file 1 [file 41467_2026_74815_MOESM1_ESM.pdf]

**Supplementary Information for**

# Magma convection favors ephemeral melt-rich bodies within mushy reservoirs

Jie Chen[1,4*], Lydéric France[2,3], Jean-Arthur Olive[1]

[1] Laboratoire de Géologie, Ecole Normale Supérieure/CNRS UMR 8538, PSL Research University, Paris 75005, France

[2] Université de Lorraine, CNRS, CRPG, Nancy, France

[3] Institut Universitaire de France (IUF), France

[4] State Key Laboratory of Submarine Geoscience; School of Oceanography, Shanghai Jiao Tong University, Shanghai 200030, China

*Corresponding author: Jie Chen (chenjie.geo@sjtu.edu.cn)

**This PDF file includes:**

- Text for model sensitivity
- Table S1
- Fig. S1-S15

**Model sensitivity**

**Numerical resolution**

We performed a resolution test, comparing a grid cell spacing of 50 m (used in the main text) with a finer resolution of 25 m (Fig. S11). The test was conducted with a maximum hydrothermal depth ($Z_H$) of 1.5 km and 6 values of $\beta$ (melt emplacement mode) ranging from 0 to 4 $m^{-1}$. We quantified the maximum thickness ($D_{max}$), area, and melt volume of the magma body, at 0.1-year intervals over a 30 kyr period following the establishment of a quasi-steady thermal regime. The results show only minor differences for $\beta$ = 0-1 $m^{-1}$ and virtually no differences for $\beta \geq 2$ $m^{-1}$, indicating that the model results are insensitive to grid resolution within this range.

**Melt sill thickness and time interval of melt injection**

We performed an additional sensitivity test varying the melt sill thickness ($H_{sill}$), comparing 50 m (used in the main text) with a thinner sill of 25 m, at the same grid cell spacing of 25 m. These configurations correspond to melt injection intervals ($\tau$) of 200 and 100 years, respectively (Fig. S12). Using the same diagnostic metrics ($D_{max}$, area, and melt volume), the results again show only minor differences for $\beta$ = 0-1 $m^{-1}$ and virtually no differences for $\beta \geq 2$ $m^{-1}$. This confirms that the main conclusions are robust with respect to reasonable variations in melt injection thickness and frequency.

**Melt viscosity**

The kinematic viscosity variability of liquid basaltic magma, $\nu_{m0}$, is assumed to be constant at 0.01 $m^2/s$ in our model, although it is inherently temperature-dependent. However, its variability is limited. The kinematic melt viscosity can be derived from the thermodynamic model Rhyolite-MELTS [1], which is the same framework used to derive the temperature-crystal fraction relationship [2] (Fig. 3A). We calibrate it to $\nu_{m0}$ at the liquidus temperature of 1250°C. Melt viscosity varies by less than one order of magnitude over the magma temperatures of 1250-1150°C (Fig. S13). In contrast, the kinematic magma viscosity ($\nu_m$), computed as a function of crystal fraction using a constant $\nu_{m0}$ at 0.01 $m^2/s$ in Equation (2), increases by up to 5 orders of magnitude as magma cools (Fig. S13). Therefore, magma viscosity is dominantly controlled by crystal fraction, and the temperature-dependent variability of melt viscosity has only a second-order effect in our model.

## Rayleigh-Nusselt scaling law

The analytical relationship between the Rayleigh number (*Ra*) and the Nusselt number (*Nu*) in convective systems is known to depend on the flow regime and fluid properties, particularly at high *Ra* and Prandtl number (*Pr*), as expected for magmatic systems. Experimental and theoretical studies have proposed different *Ra-Nu* power-law scaling across distinct *Ra–Pr* regimes [3,4]. The classical *Ra-Nu* scaling suggests $Nu \sim Ra^{1/3}$ in a high *Ra* and *Pr* regime, which is adopted in the main text. Alternatively, a linear combination of the 1/4 and the 1/3 power laws:

$$Nu = 0.27Ra^{1/4} + 0.038Ra^{1/3}, \tag{1}$$

effectively mimics $Nu \sim Ra^{2/7}$ scaling over a broad range of *Ra* spanning as large as ten orders of magnitude [3].

To evaluate the sensitivity of our results to the choice of the *Ra-Nu* scaling, we performed an additional test by varying the power law exponents of 1/3 and 2/7 in Equations (4) and (13).

$$Nu = (Ra/Ra_c)^{2/7}, \tag{2}$$

Again, using the same diagnostic metrics ($D_{max}$, area, and melt volume of magma body), the results show slight higher values for $\beta$ = 0-1 m⁻¹ and virtually no differences for $\beta \geq 2$ m⁻¹ (Fig. S14). These differences are minor and do not alter the overall trends in the dynamics of the magma body, which, therefore, confirms that our main conclusions are robust with respect to reasonable variations in the *Ra-Nu* scaling law.

## Critical crystal fraction between magma and mush

We performed sensitivity tests on the critical crystal fraction ($X_c$), comparing values of 0.55, 0.6 (used in the main text), and 0.65 (Fig. S15). For a given $\beta$, all three diagnostic metrics ($D_{max}$, area, and melt volume) increase with increasing $X_c$. The differences are substantial at low $\beta$, but become negligible for $\beta \geq 2$ m⁻¹.

| Symbol | Definition | Value | Units |
|---|---|---|---|
| $x$ | Horizontal model distance from the ridge axis | 8000 | m |
| $z$ | Model depth below the seafloor | 6000 | m |
| $\varphi$ | Lower-crustal melt flux for the half plate at 9°N of East Pacific Rise | 250 | $m^2$/kyr |
| $W_{sill}$ | Width of melt sill | 1000 | m |
| $H_{sill}$ | Thickness of melt sill | 50 | m |
| $\tau$ | Time interval of melt emplacement | 200 | year |
| $\beta$ | Decay rate of depth distribution of melt emplacement (Equation 1) | 0-4 | $m^{-1}$ |
| $Z_{AML}$ | Depth of the topmost seismically-imaged axial melt lense | 1500 | m |
| $Z_{Moho}$ | Depth of Moho | 6000 | m |
| $T_S$ | Basaltic solidus temperature | 900 | °C |
| $T_L$ | Basaltic liquidus temperature | 1250 | °C |
| $T_c$ | Critical temperature between magma and mush | 1150 | °C |
| $\Delta T$ | Temperature difference between magma and surrounding mush | - | °C |
| $D$ | Thickness of magma body | - | m |
| $D_{max}$ | Maximum thickness of magma body | - | m |
| $X$ | Crystal fraction | - | |
| $X_c$ | Critical crystal fraction between magma and mush | 0.6 | |
| $X_{min}$ | Minimum crystal fraction of the magma body | - | |
| $L$ | Latent heat of crystallization | $4.2\times10^5$ | J/kg |
| $\rho_M$ | Magma density | 2700 | $kg/m^3$ |
| $Z_H$ | Maximum potential depth of hydrothermal system | 0, 1.5, 3 | km |
| $T_H$ | Maximum temperature of hydrothermal system | 500 | °C |
| $k(x,z)$ | Permeability | - | $m^2$ |
| $k_{max}$ | Axial maximum permeability at the seafloor ($x$ = 0 and $z$ = 0) | $10^{-14}$ | $m^2$ |
| $k_0(x)$ | Seafloor permeability ($z$ = 0) | $10^{-15}$-$10^{-14}$ | $m^2$ |
| $P_d$ | Normalized pressure sensitivity coefficient | 10-30 | MPa |
| $P_{eff}$ | Effective pressure | - | MPa |
| $\rho_r$, $\rho_f$ | Density of solid rock and hydrothermal fluid | 2900/~400 | $kg/m^3$ |
| $C_r$, $C_f$ | Heat capacity of solid rock and hydrothermal fluid | 1000/4000 | W/kg/°C |
| $\nu_{m0}$ | Magma viscosity at $T_L$ | 0.01 | $m^2/s$ |
| $\nu_m$ | Magma viscosity | - | $m^2/s$ |
| $\lambda$ | Thermal conductivity | 3 | W/m/°C |
| $Nu$ | Magmatic Nusselt number | - | |
| $Ra$ | Magmatic Rayleigh number | - | |
| $Ra_c$ | Critical Rayleigh number | $10^3$ | |
| $\alpha_m$ | Thermal expansion coefficient | $5\times10^{-5}$ | °$C^{-1}$ |
| $a_m$ | Thermal diffusivity | $8\times10^{-7}$ | $m^2/s$ |
| $g$ | Acceleration of gravity | 9.8 | $m/s^2$ |
| $P_0$ | Pressure at seafloor | 20 | MPa |
| $T_0$ | Temperature at seafloor | 0 | °C |
| $T_b$ | Bottom boundary temperature | 1100-800 | °C |
| $\phi$ | Porosity | 0.03 | |

Table S 1. Summary of parameters in numerical models.

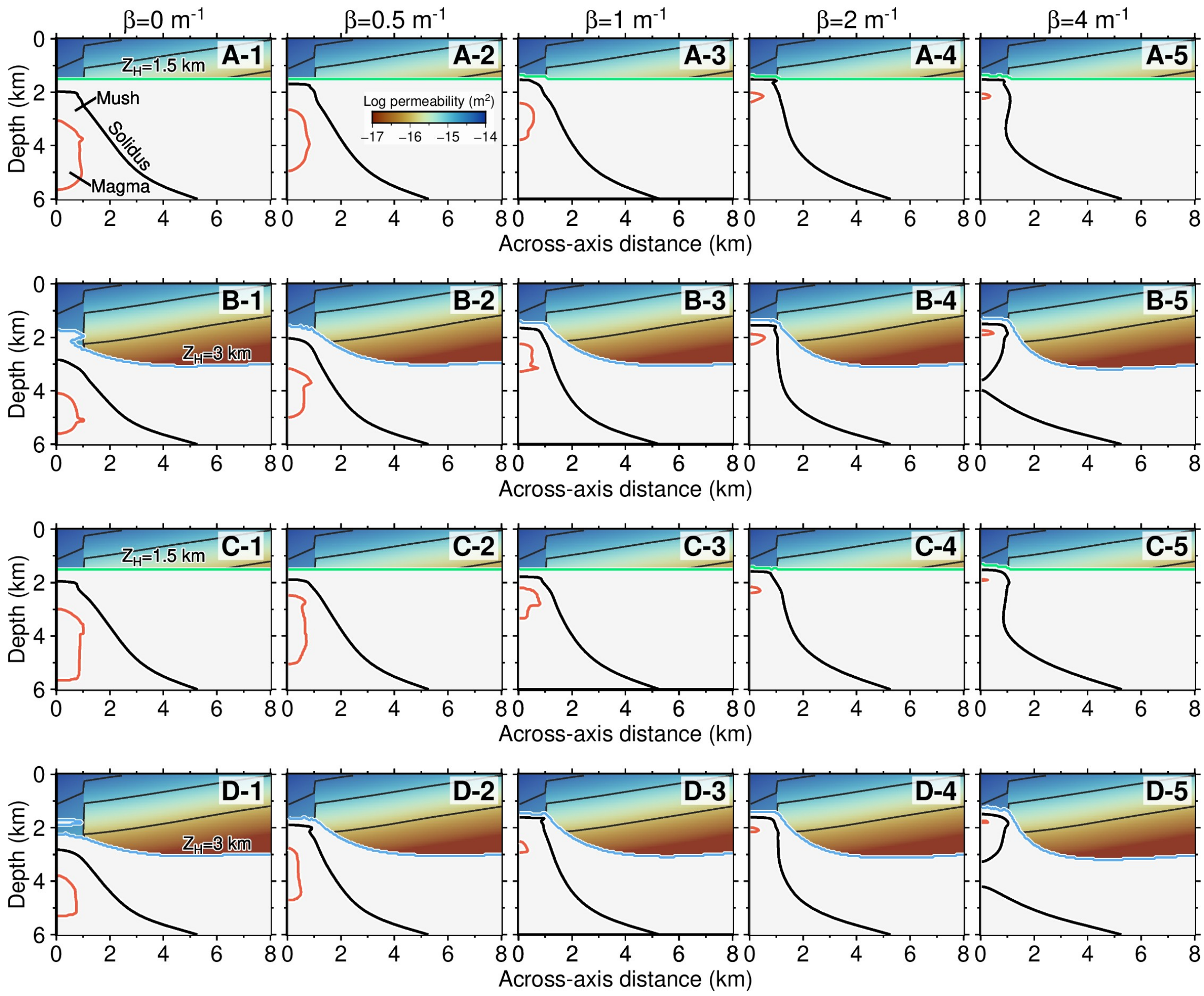


Figure S 1 | **Permeability structure.** (A) and (B) No-magma-convection simulations at ZH=1.5 and 3 km, corresponding to Fig. 2E and 2F, respectively. (C) and (D) Magma-convection simulations at ZH=1.5 and 3 km, corresponding to Fig. 4E and 4F, respectively.

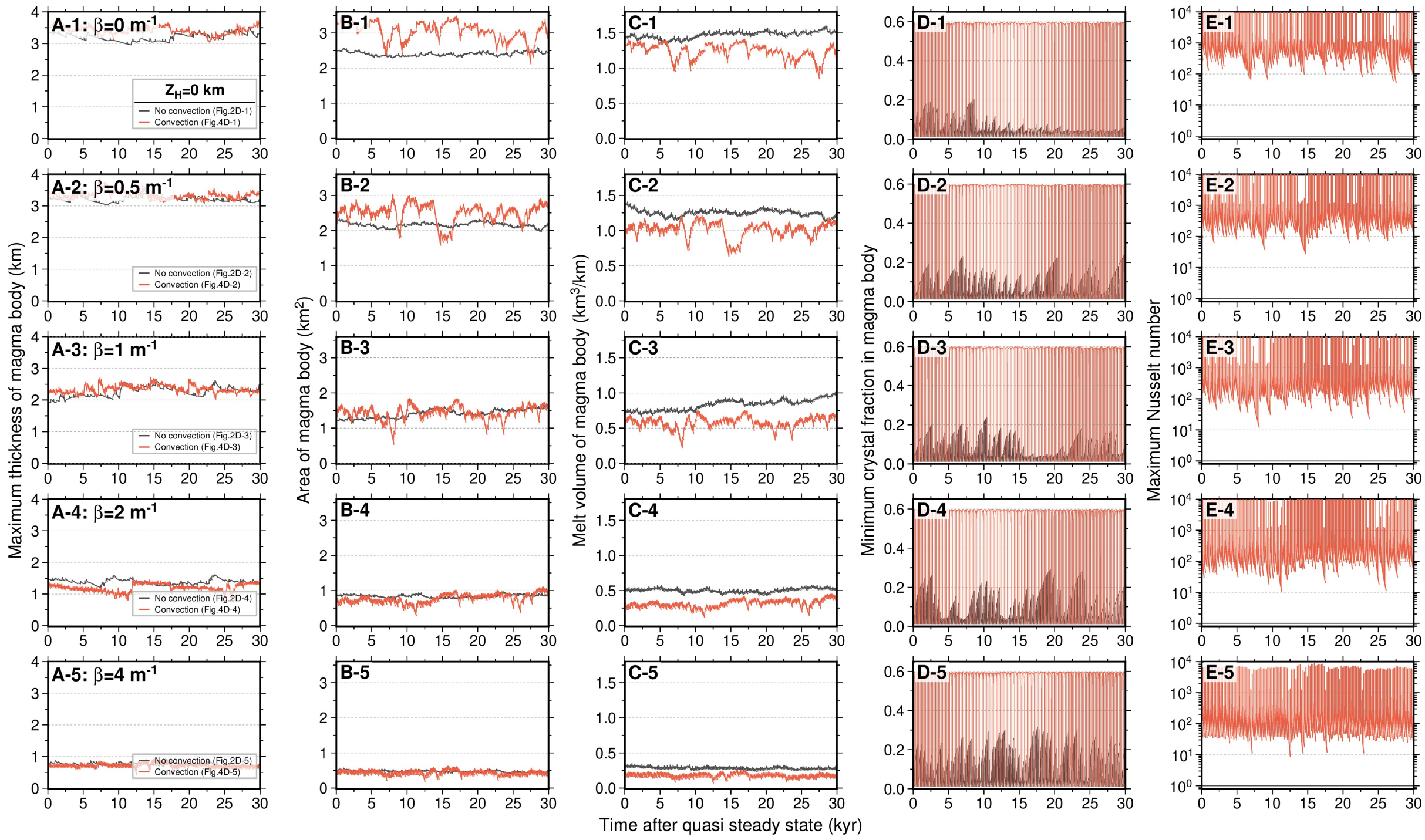


Figure S 2 | **Temporal evolution of the magma body after reaching quasi steady state over 30 kyrs at $Z_H$ = 0.** All are measured every 0.1 year over a 30 kyrs (i.e., 300 k measurements). $\beta$ increases from 0 to 4 $m^{-1}$, comparing models with (red lines) and without (black lines) magma convection. (A) Maximum thickness. (B) Area. (C) Melt volume. (D) Minimum crystal fraction. (E) Maximum Nusselt number.

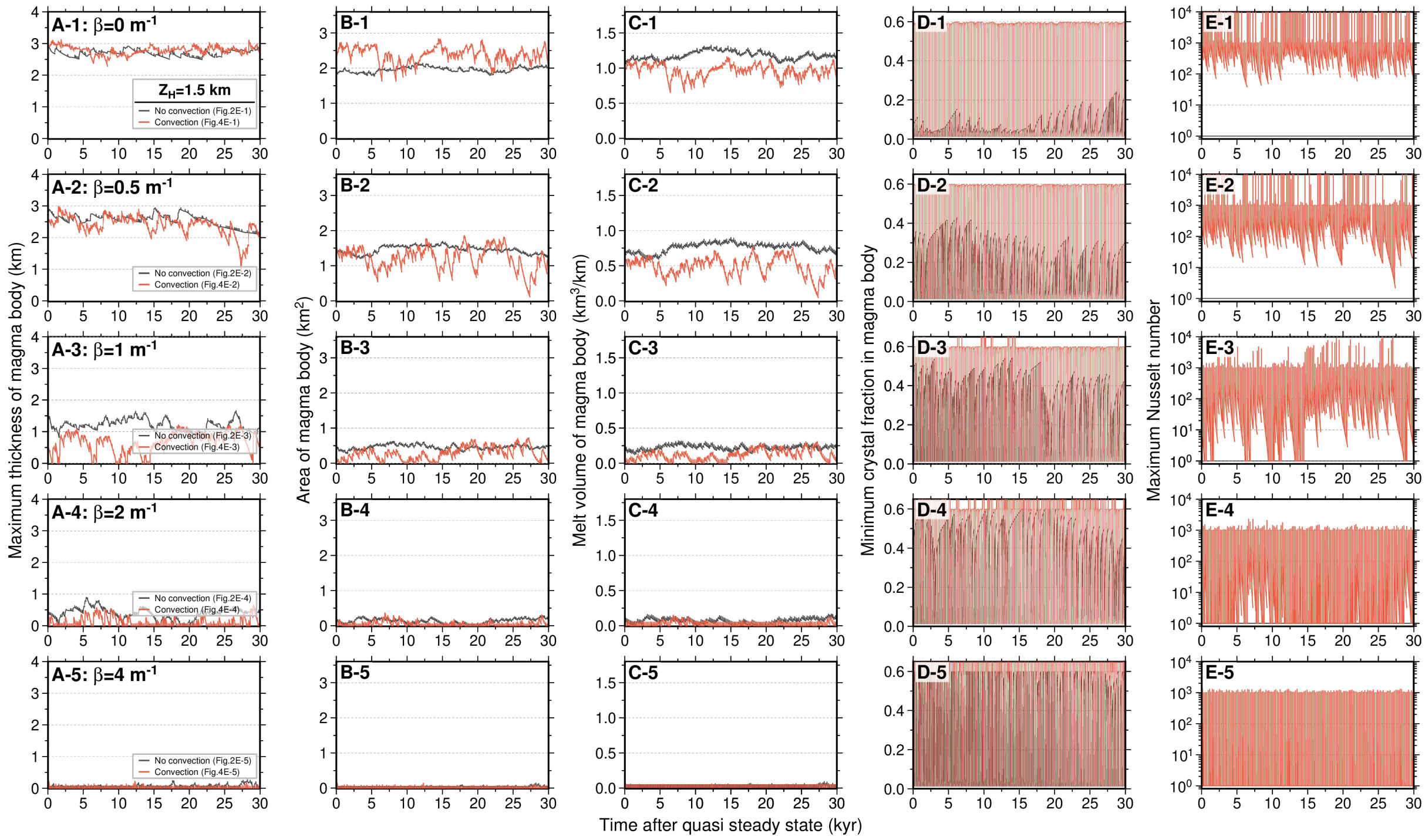


Figure S 3 | **Temporal evolution of the magma body after reaching quasi steady state over 30 kyrs at $Z_H$ = 1.5 km.**

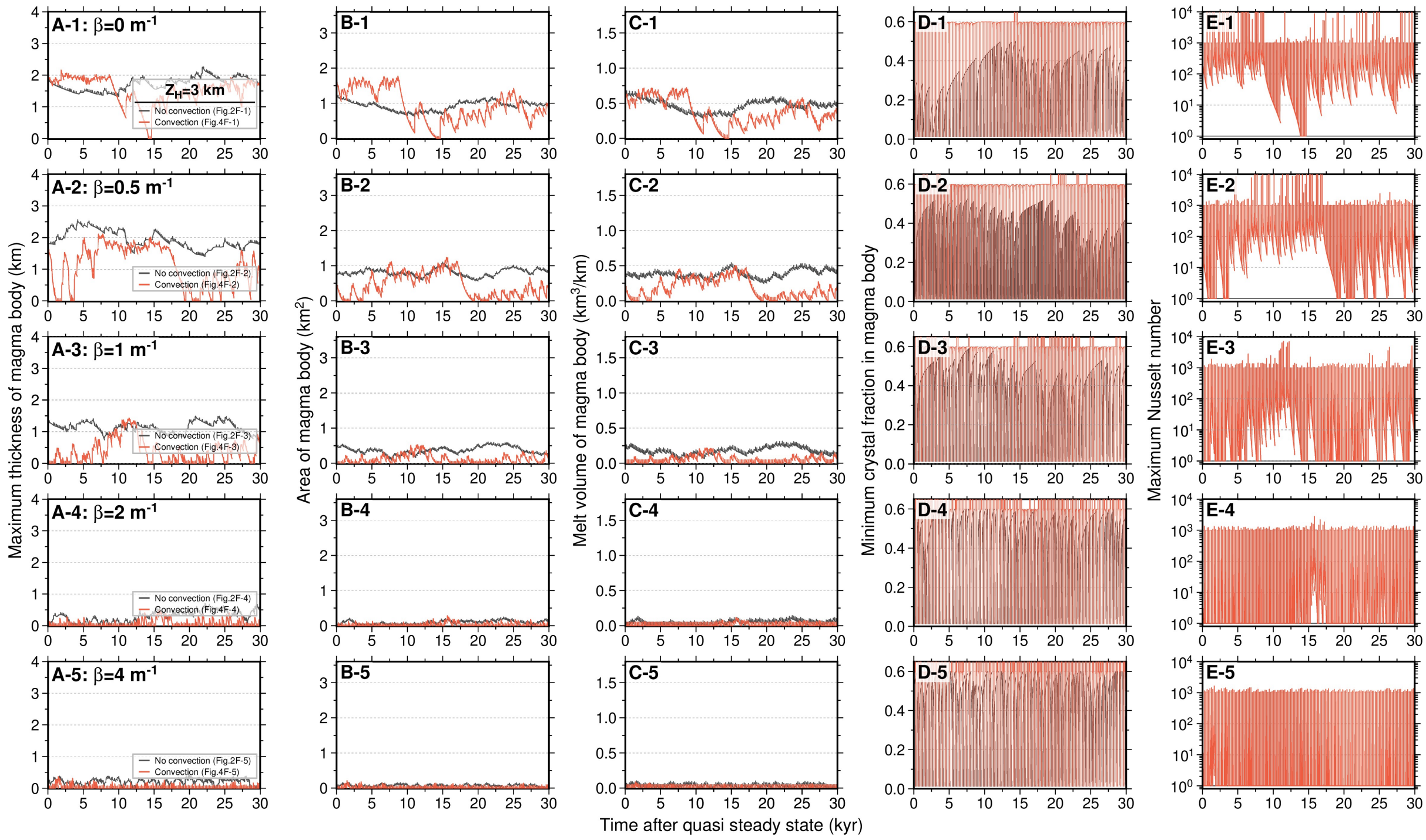


Figure S 4 | **Temporal evolution of the magma body after reaching quasi steady state over 30 kyrs at $Z_H$ = 3 km.**

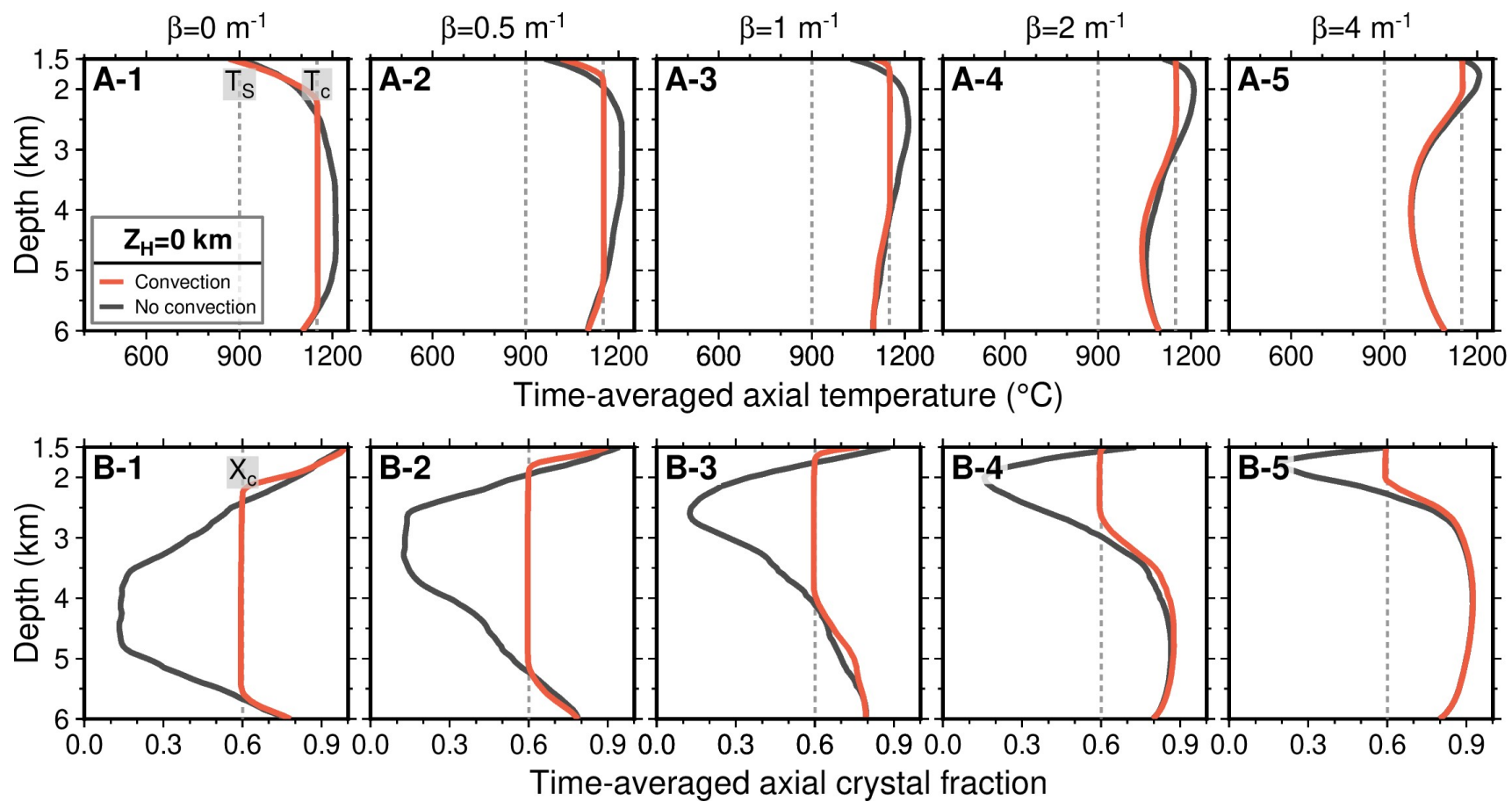


Figure S 5 | **Time-averaged axial profiles of temperature (A) and crystal fraction (B) over 30 kyrs at $Z_H$ = 0.** $\beta$ increases from 0 to 4 m$^{-1}$, comparing models without (black lines) and with (red lines) magma convection and corresponding to models in Fig. 2D and Fig. 4D.

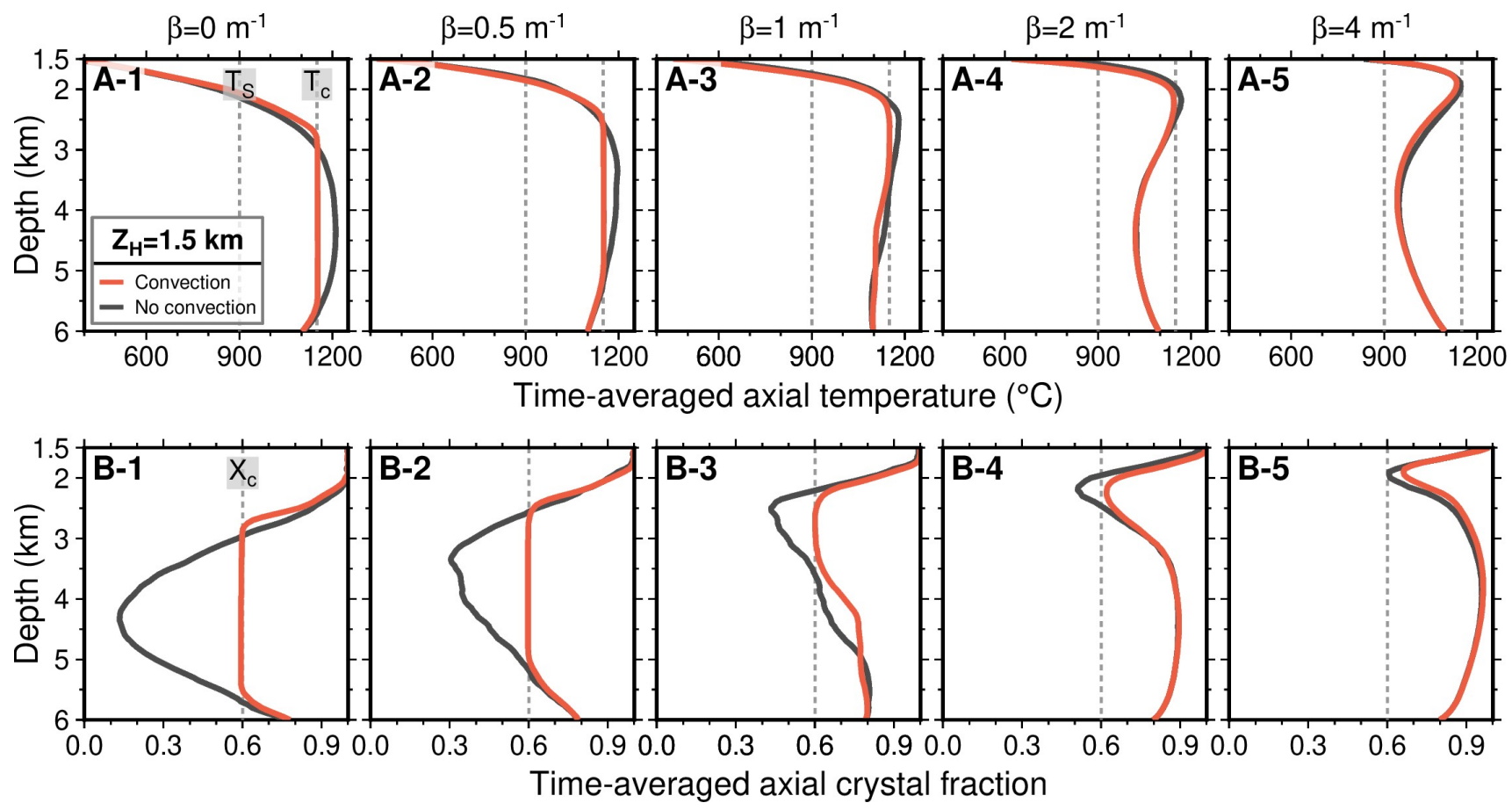


Figure S 6 | **Time-averaged axial profiles of temperature (A) and crystal fraction (B) over 30 kyrs at $Z_H$ = 1.5 km.** Profiles correspond to models in Fig. 2E and Fig. 4E.

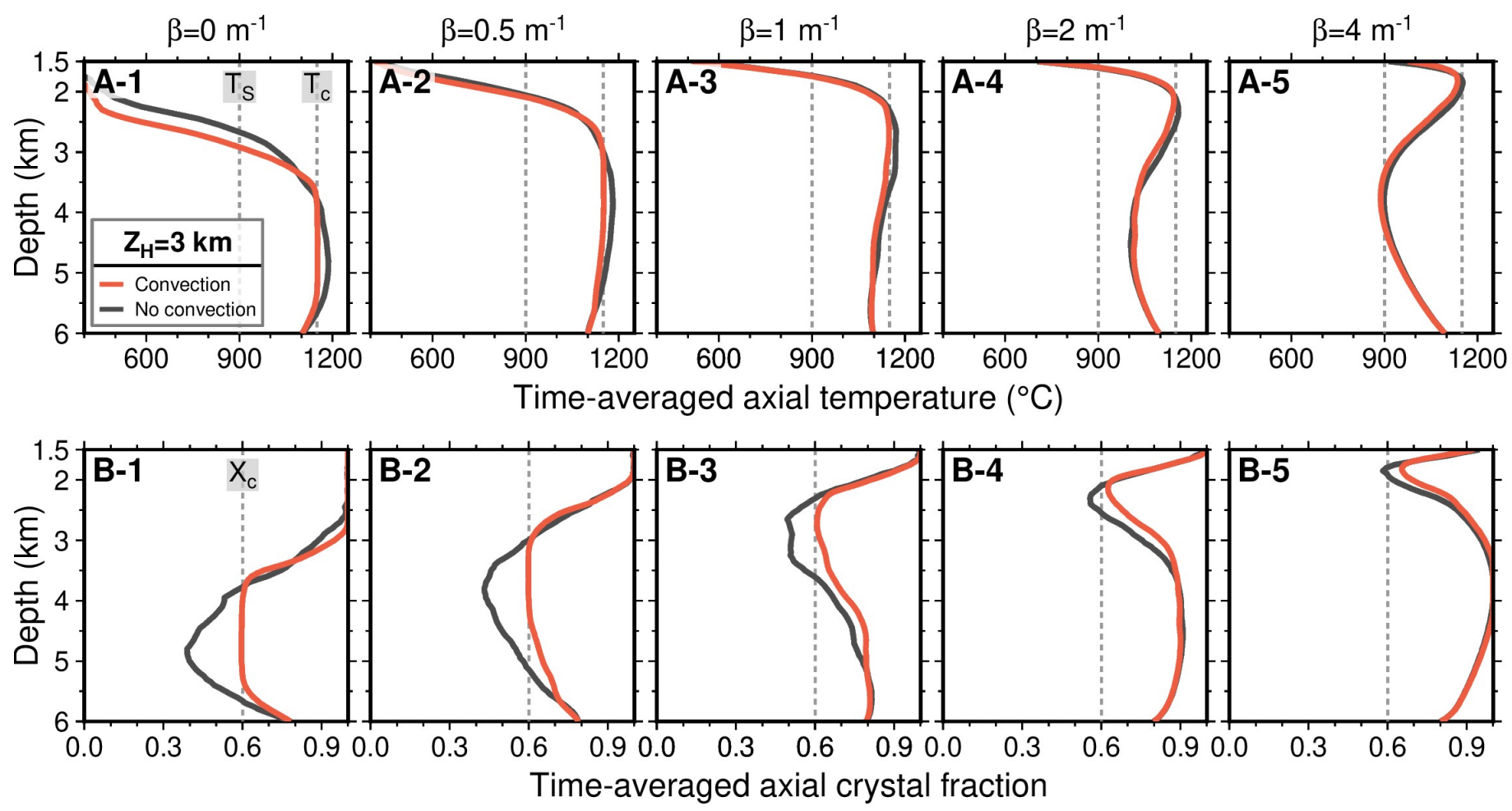


Figure S 7 | **Time-averaged axial profiles of temperature (A) and crystal fraction (B) over 30 kyrs at $Z_H$ = 3 km.** Profiles correspond to models in Fig. 2F and Fig. 4F.

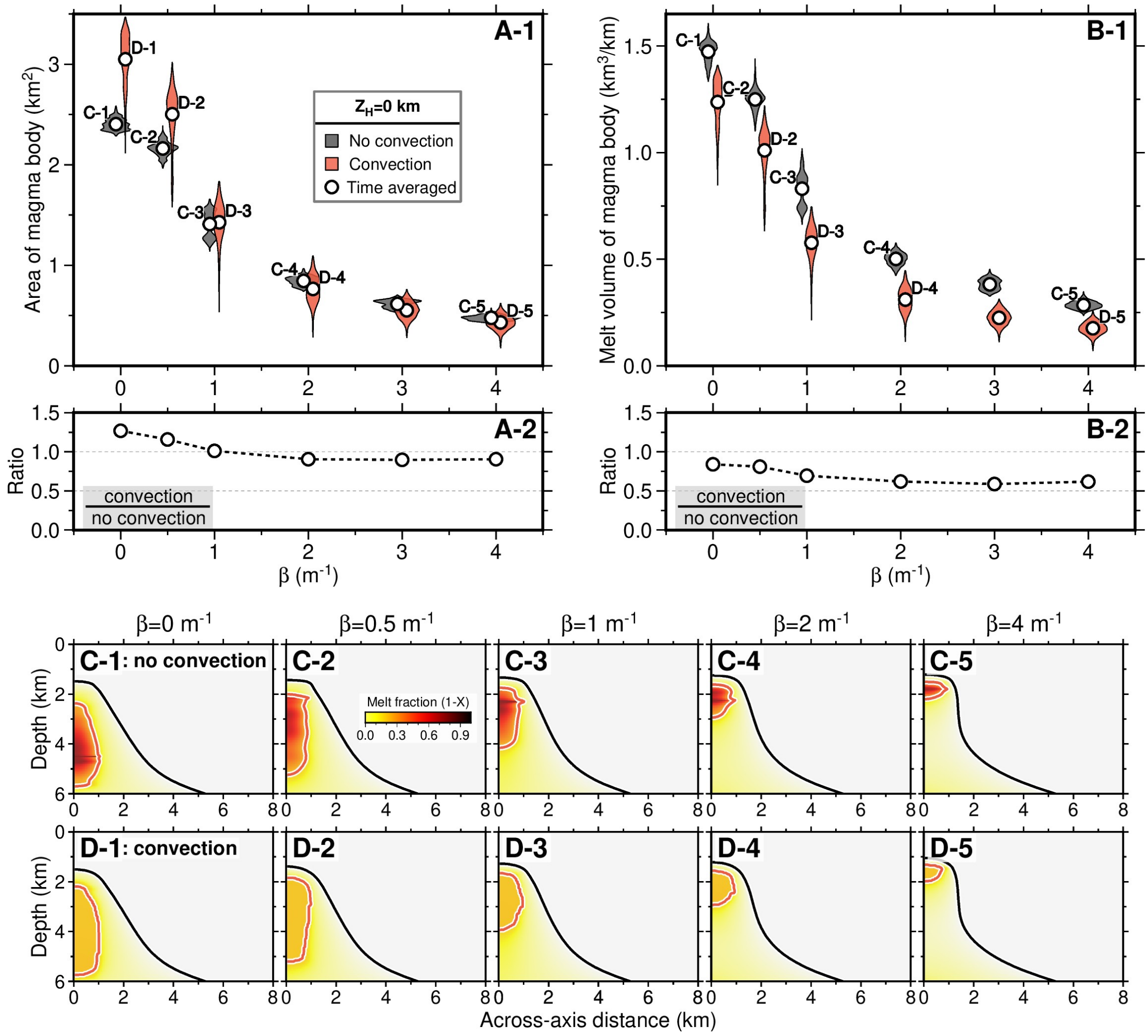


Figure S 8 | **Comparison of simulations with and without magma convection at $Z_H$ = 0.** See legend in Fig. 5. Melt-fraction snapshots correspond to temperature snapshots in Fig. 2D and Fig. 4D, respectively.

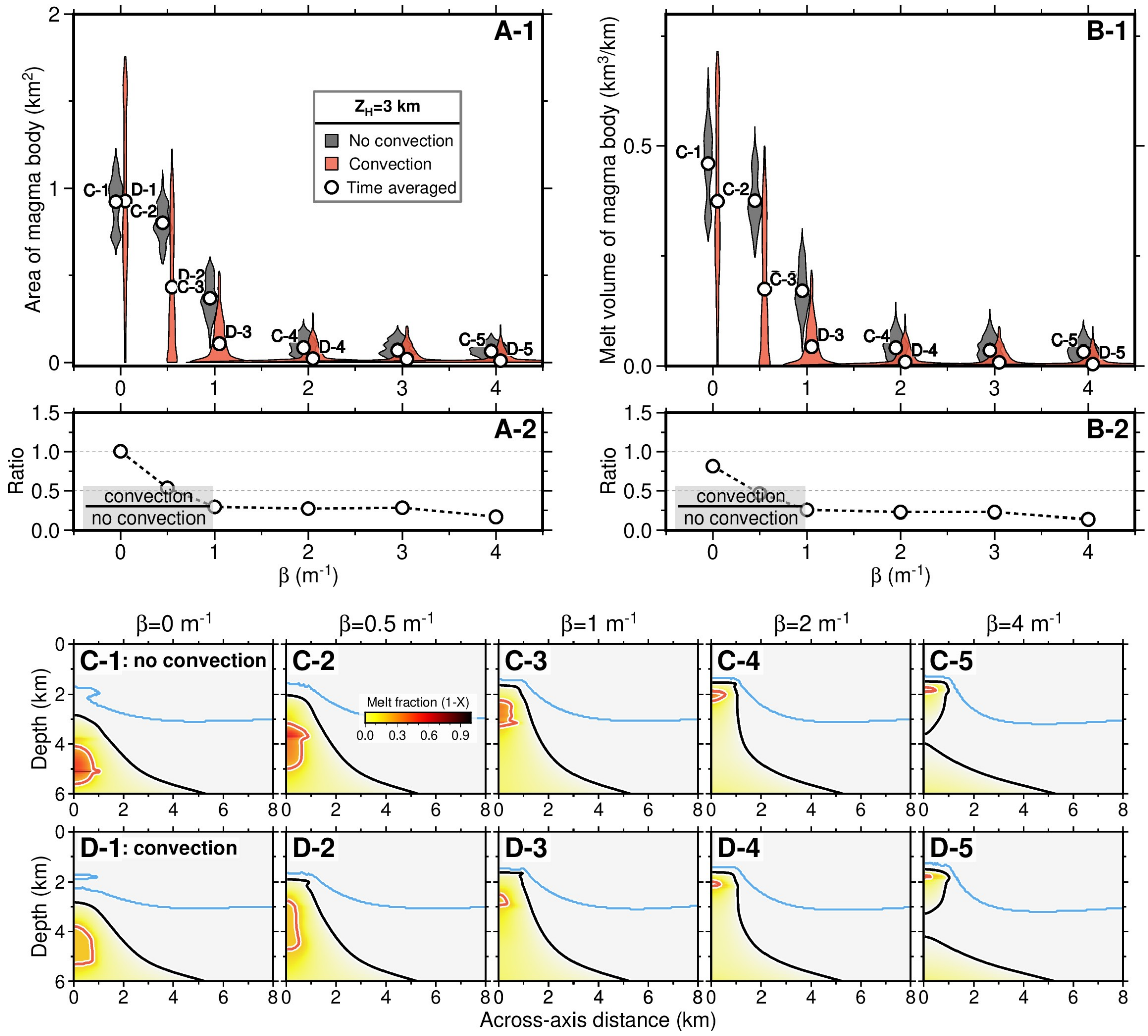


Figure S 9 | **Comparison of simulations with and without magma convection at $Z_H$ = 3 km.** See legend in Fig. 5. Melt-fraction snapshots correspond to temperature snapshots in Fig. 2F and Fig. 4F, respectively.

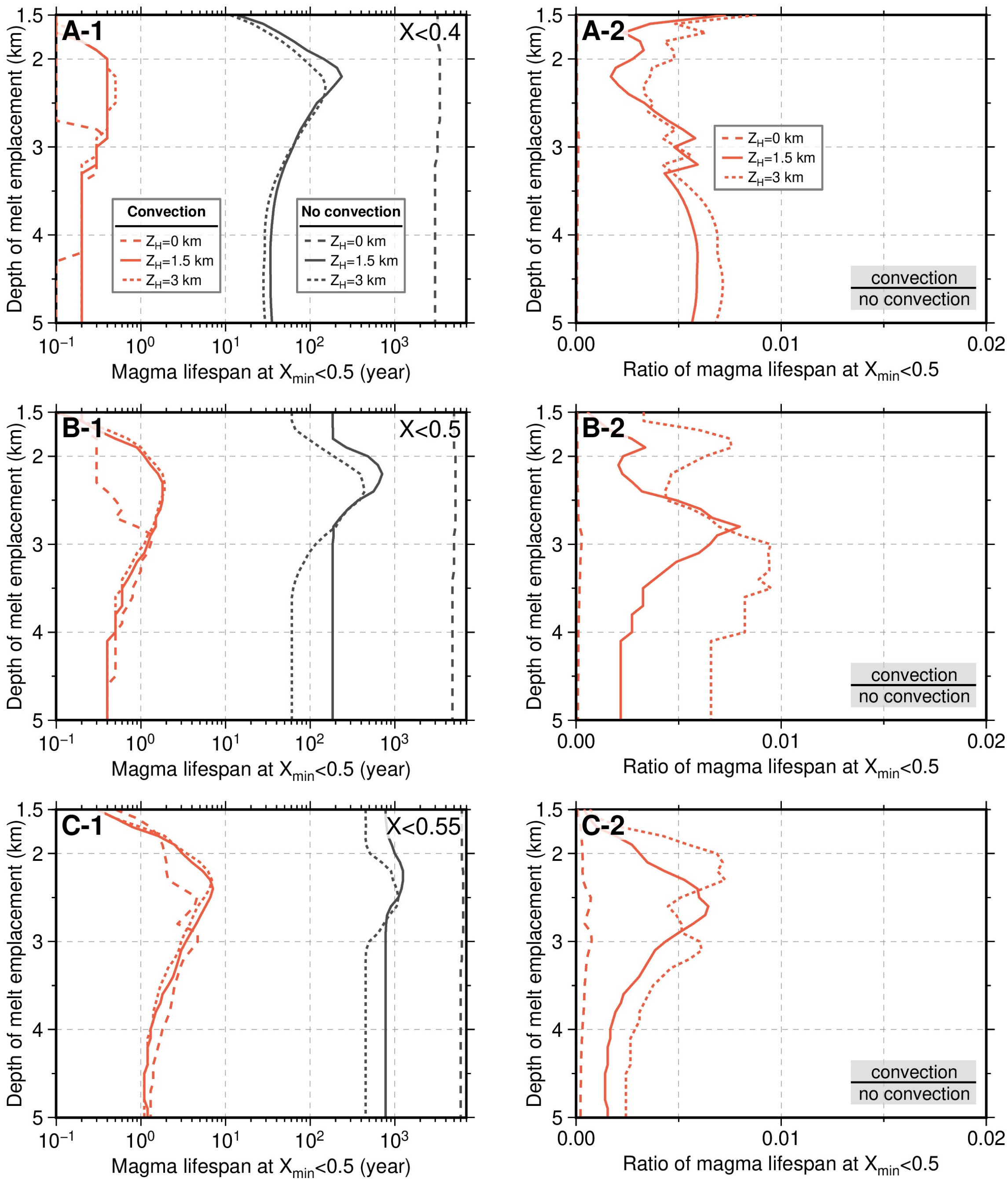


Figure S 10 | **Magma lifespan (left panels) and ratio of magma lifespan with convection to no convection (right panels) at depths of 1.5-5 km, using thresholds of minimum crystal fraction at 0-0.4 (A), 0-0.5 (B), and 0-0.55 (C).**

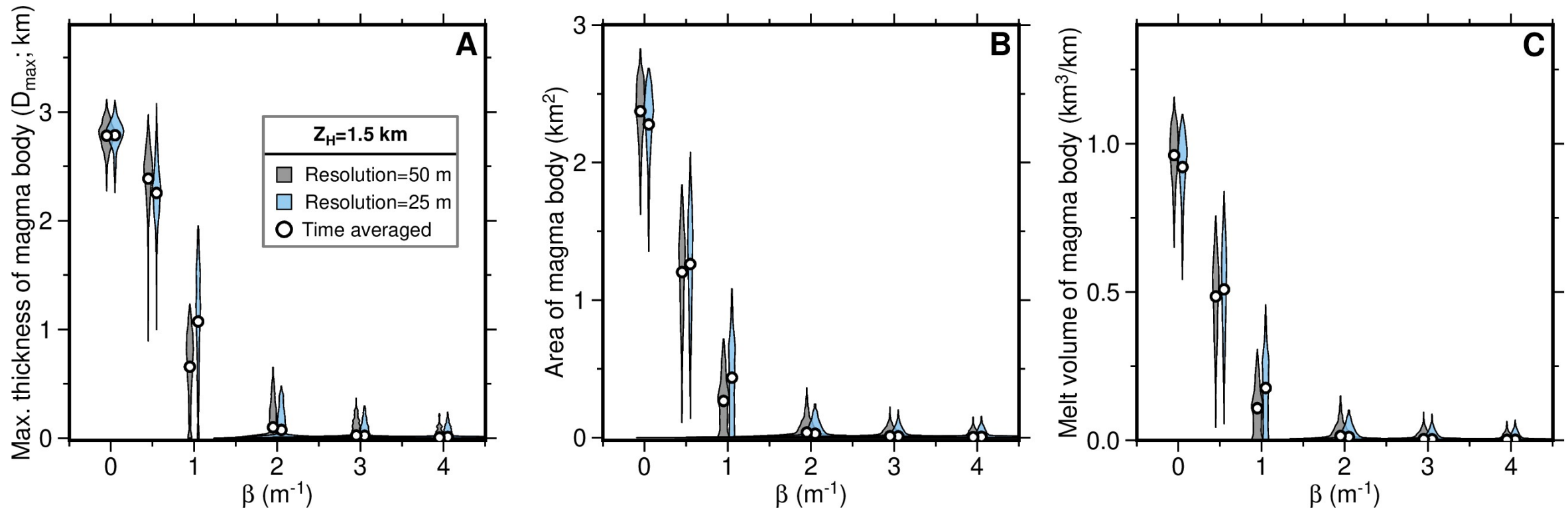


Figure S 11 | **Sensitivity test of model resolution (50 m vs. 25 m).**

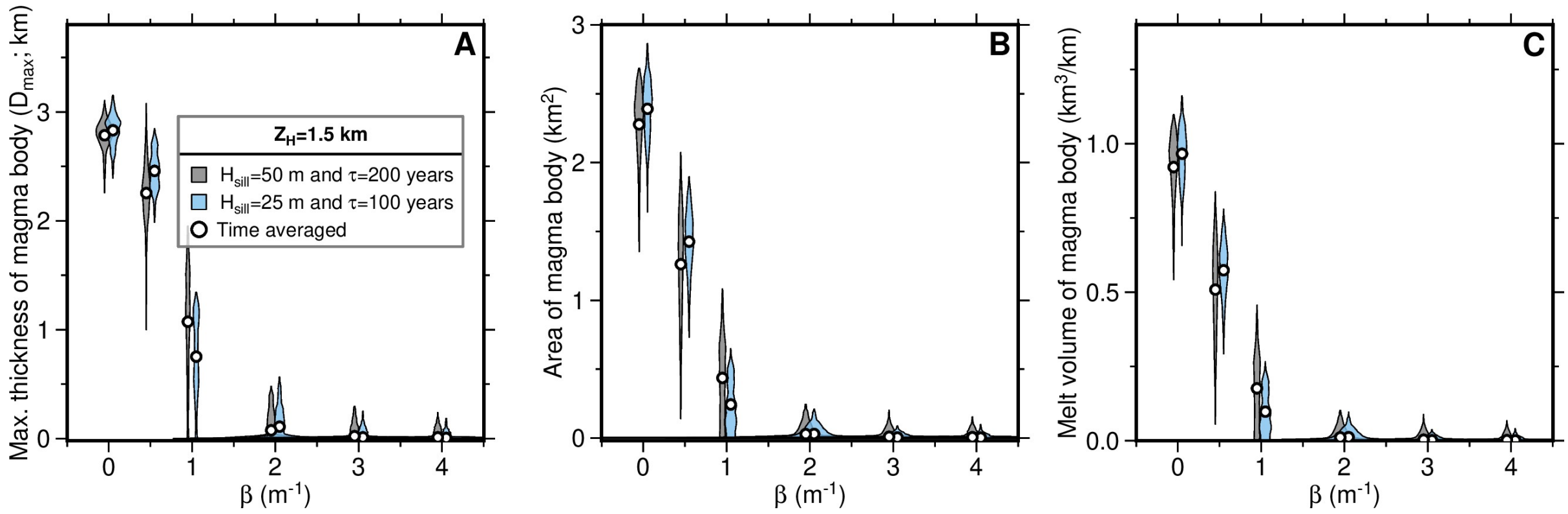


Figure S 12 | **Sensitivity test of melt sill thicknesses (*$H_{sill}$*; 50 m vs. 25 m) and the corresponding time intervals of melt injection (*τ*; 200 years vs. 100 years), at the same grid cell spacing of 25 m.**

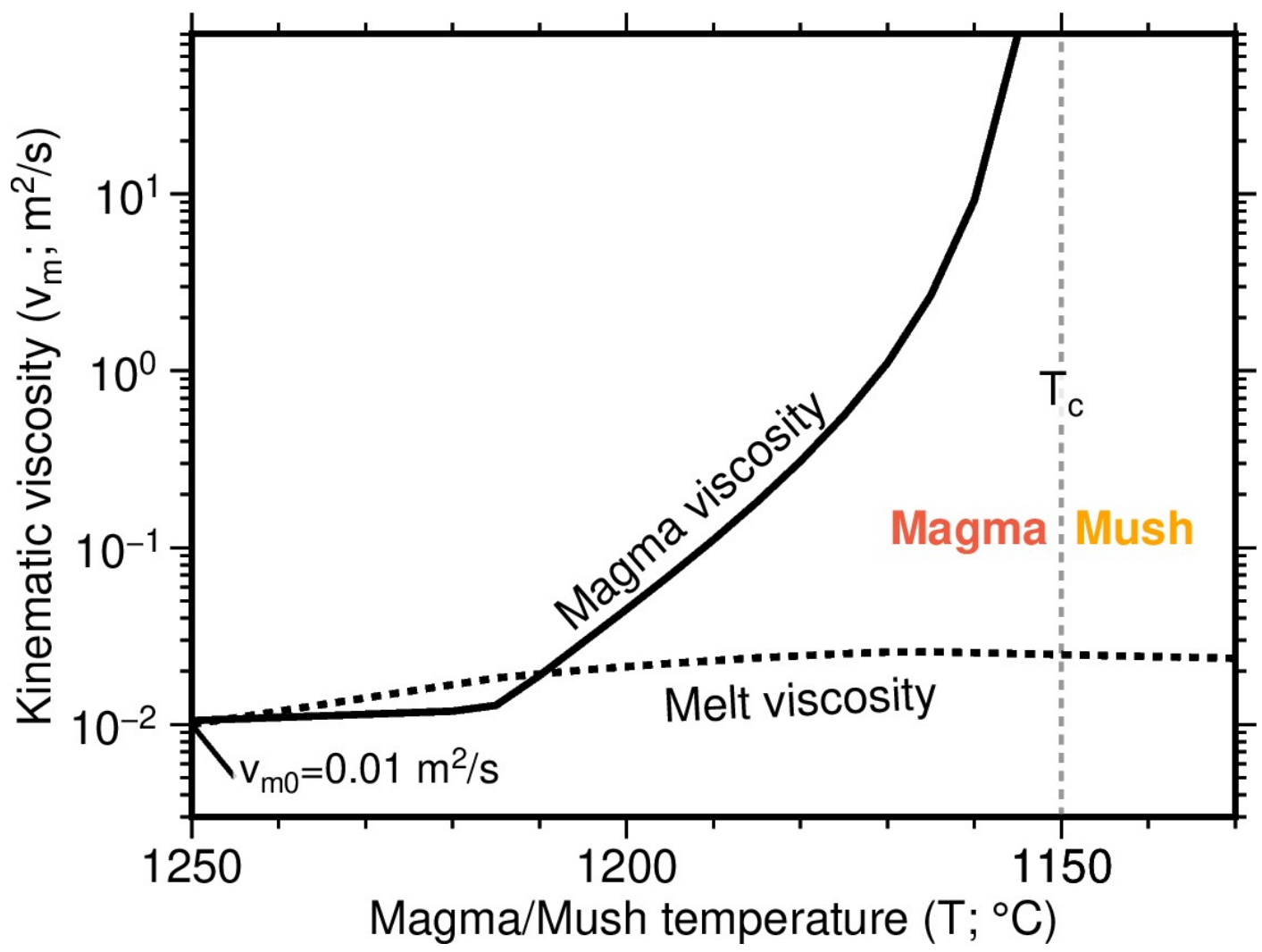


Figure S 13 | **Magma and melt kinematic viscosity verses temperatures.** [2]

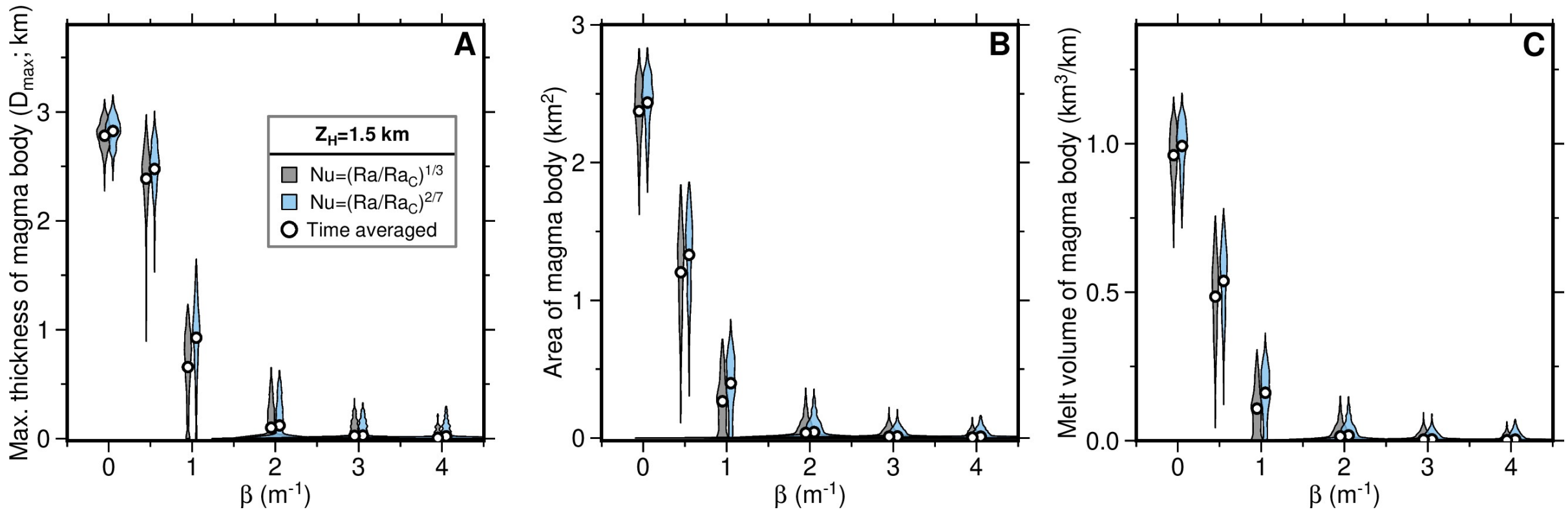


Figure S 14 | **Sensitivity test of Rayleigh (*Ra*) – Nusselt (*Nu*) scaling laws, $Nu=(Ra/Ra_c)^{1/3}$ vs. $Nu=(Ra/Ra_c)^{2/7}$.**

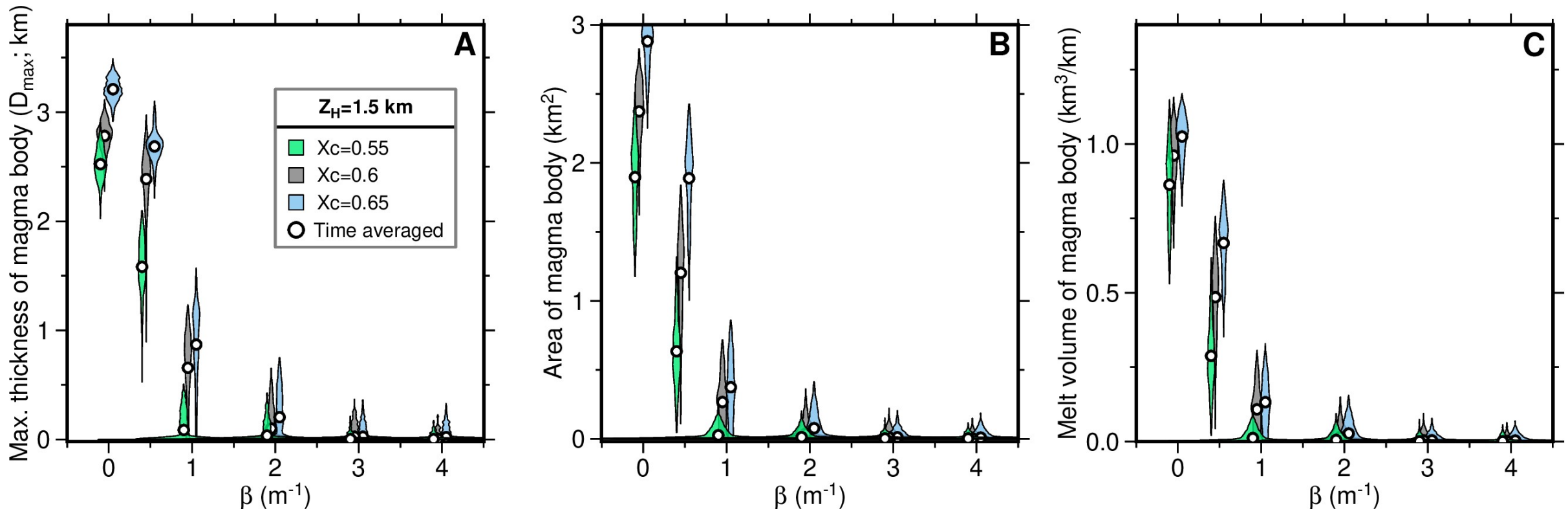


Figure S 15 | **Sensitivity test of critical crystal fraction ($X_c$) at 0.55, 0.6 (used in the main text), and 0.65.**

## References


1. Toussaint, A. & France, L. The importance of considering temperature- and composition-dependent partition coefficients in differentiation models. *Lithos* **522–523**, 108395 (2026).
2. France, L., Charvet, V. & Toussaint, A. Advances in igneous petrology: Coupling chemical maps with thermodynamic models to tackle mush crystallization dynamics and crystal–melt segregation. *J. Petrol.* **66**, egaf044 (2025).
3. Grossmann, S. & Lohse, D. Scaling in thermal convection: a unifying theory. *J. Fluid Mech.* **407**, 27–56 (2000).
4. Grossmann, S. & Lohse, D. Thermal Convection for Large Prandtl Numbers. *Phys. Rev. Lett.* **86**, 3316–3319 (2001).
