# Supplementary material for: Magma convection favors ephemeral melt-rich bodies within mushy reservoirs
Source: Nat Commun. 2026 Jun 25;17:7957. doi: 10.1038/s41467-026-74815-1 (PMC13448659; doi:10.1038/s41467-026-74815-1)
Supplement: Supplementary file 2 [file 41467_2026_74815_MOESM2_ESM.pdf]

nature portfolio

Peer Review File

# Magma convection favors ephemeral melt-rich bodies within mushy reservoirs

Corresponding Author: Dr Jie Chen

**This file contains all reviewer reports in order by version, followed by all author rebuttals in order by version.**

Version 0:

Reviewer comments:

Reviewer #1

(Remarks to the Author)
Here's a polished version of your review with grammar, wording, and consistency cleaned up, but keeping your structure and arguments.

---

The manuscript presents 2-D numerical simulations of a lower-crustal magma–mush system beneath a fast-spreading ridge. The authors build on an existing thermo-mechanical framework in which sill-like melt lenses are repeatedly emplaced into a gabbroic mush between the axial magma lens (AML) and the Moho. Heat is removed by conduction in the crust, by parameterized convection within melt-rich regions (via a Nusselt-number enhancement of thermal diffusivity), and by Darcy-style hydrothermal circulation in a permeable upper layer. The main quantitative result is that including convective heat transport in the magma lenses reduces the lifetime of "mobilizable" melt from hundreds to thousands of years (conduction-only) to at most a few years, while leaving the long-term thermal structure of the mush largely unchanged.

The physical interpretation of the process is correct, but not novel, and its implementation within the mathematical model requires further clarification and adjustment.

---

Novelty

1. The role of convective heat transfer during the emplacement of magma into the crust has been well understood since the classical papers by Huppert and Sparks (1988):
Huppert, H.E., Sparks, R.S.J., 1988. Melting the roof of a chamber containing a hot, turbulently convecting fluid. Journal of Fluid Mechanics 188, 107–131. [https://doi.org/10.1017/S0022112088000655](https://doi.org/10.1017/S0022112088000655)
Huppert, H.E., Sparks, R.S.J., 1988. The Generation of Granitic Magmas by Intrusion of Basalt into Continental Crust. Journal of Petrology 29, 599–624. [https://doi.org/10.1093/petrology/29.3.599](https://doi.org/10.1093/petrology/29.3.599)

2. The parameterization of effective heat transfer in convective regions was already implemented in, e.g., Annen et al. (2008), where a temperature-averaging procedure was applied to magma-rich regions.
Annen, C., Pichavant, M., Bachmann, O., Burgisser, A., 2008. Conditions for the growth of a long-lived shallow crustal magma chamber below Mount Pelée volcano (Martinique, Lesser Antilles Arc). Journal of Geophysical Research: Solid Earth 113. [https://doi.org/10.1029/2007JB005049](https://doi.org/10.1029/2007JB005049)

3. Recent papers by Gerbault, Borisova and Melnik introduced an effective heat capacity based on a Nu–Ra parameterization. In the 2025 paper, the simplified 1-D model was compared with a full-scale 2-D numerical simulation.
Gerbault, M., Melnik, O., Borisova, A., 2025. Numerical simulation of magma-rock interaction at Krafla volcano using OpenFOAM software and a simplified thermal model. Solid Earth 16, 1307–1333. [https://doi.org/10.5194/se-16-1307-2025](https://doi.org/10.5194/se-16-1307-2025)
Borisova, A.Y., Melnik, O.E., Gaborit, N., Bindeman, I.N., Traillou, T., Raffarin, M., Stefánsson, A., Laurent, O., Leisen, M.,

Llovet, X., de Parseval, P., Proietti, A., Tait, S., 2023. In situ probing of the present-day zircon-bearing magma chamber at Krafla, Northeastern Iceland. Frontiers in Earth Science 11.

4. A relevant model by Liu & Buck (2023) is not discussed in the text.
Liu, Z., Buck, W.R., 2023. The spreading rate dependence of the distribution of axial magma lenses along mid-ocean ridges. Proceedings of the National Academy of Sciences 120, e2214048120. [https://doi.org/10.1073/pnas.2214048120](https://doi.org/10.1073/pnas.2214048120)

The model

The model is not well discussed in the paper, and there is also no complete description in the referenced papers. All governing equations should be clearly specified in the Supplementary Material.

1. It is not clear which equations are solved. Is it only the heat equation, or a full advection–diffusion equation? How are the displacements of the magma and mush treated following sill injection?

2. How is the thermal conductivity parameterized? Only Nu(Ra) is discussed in the text.

3. How is the Rayleigh number calculated? It depends on the temperature difference and the characteristic vertical extent of the domain (Ra ~ deltaT D^3). How is deltaT defined? As the sill cools down and the mush heats up, the temperature difference progressively decreases. Is D fixed? What happens when mobile magma batches merge?

4. Gerbault et al. have shown that the main driver for convection can be compositional rather than purely thermal, because densities of melt and crystals for basalt differ significantly. The compositional Rayleigh number is more than an order of magnitude larger than the thermal one. This dependence should be taken into account.

5. It is not clear why the viscosity depends only on the crystal content. Melt viscosity itself is strongly temperature-dependent.

In conclusion, the paper is currently not suitable for publication in Nature Communications. A major revision is required to clarify the model, properly position the work with respect to existing literature, and address the issues listed above.

(Remarks on code availability)

Reviewer #2

(Remarks to the Author)
This contribution is a nice build up on previous work from the same group that focusses on the specific question of the lifespan of eruptible magmas below shallow magma bodies, especially below oceanic spreading ridges. The authors consider a shallow 6 km deep magmatic system at the base of which lenses of partially molten material is injected every 200 yrs, and model how coupled hydrothermal assumptions manage to explain how these lenses emplace at ca 1.5 km depth and can last several years to decades, depending on basically 2 assumptions of the initial partial melt distribution within the crustal column and the efficiency (depth) of the overlying hydrothermal system in diffusing temperature. The text is well written and figures and explanations are relatively clear, therefore a majority of my comments below are simple requests to clarify some formal explanations and descriptions.
In order for the reading audience to better grasp the novelty of the approach and the key results, I suggest to slightly reorganise the introduction section so as to make it clearer what is novel view point here with respect to previous studies (most of which by the same group).
Then I have 2 main remarks regarding the conclusions, 1) I find far-fetched to state that those results are applicable to continental settings, while no references nor quantitative arguments are brought here to support the validity of this extrapolation. So either delete that statement or feed in more quantitative demonstration. 2) the conclusion that these models explain the self-consistent pulsating behavior of shallow magmatic systems is also far fetched given that the authors consider periodical magmatic input in their models already (every 200 years) , so self-consistency is not totally demonstrated here, nevertheless they do show neatly which controlling parameters allow to reduce this basal input frequency by 2 orders of magnitude (eg. years).

I hope that the authors will be willing to make these little text adjustments in order for the reader to better understand the true added value of this contribution to our understanding of shallow magma systems dynamics. Then obviously, in my opinion it will well deserve publication.

INTRODUCTION

The first two paragraphs are well informative, but an additional sentence would be useful, that somehow would state more explicitly what your previous contributions (at least 4) or those of others have brought in terms of new insight and what you propose here as additional insight, because somehow it is not clear how by using the same numerical method, you are going to look here at a slightly different aspect, or setup or time-scale, that will more specifically answer the question of the

lifespan of eruptible magmas below shallow magma bodies. One or two sentences should be sufficient. This goes with the flaws of such publications that we need to go way back to previous authors contributions to understand the progression in developments and gained insight, it is heavy for the reader that does not necessarily follow the subject closely.

Beginning of second paragraph you broaden the contextual application of shallow magma bodies to spreading ridges, hotspots and volcanic arcs. You may add la Réunion to Hawaii which is not a unique hotspot, and Andean or other volcanic arcs and not just Cascadia. Convection in shallow volcanic arc magma bodies have also been studied in for instance Chilean plutons eg. Gutierrez et al. (2013) I find a bit restrictive to only mention well known features from the Northern America community with respect to other volcano-tectonic settings worldwide.
Second paragraph again, you may mention in the context of isolated transient and shallow magma bodies, the recent study by Gerbault et al., 2025 who modelled convection and heat transfer within a 2km deep shallow sill below Krafla, over 35 to 300 yrs. This goes with the question raised, l.59-61, of how widespread magma bodies « live » with respect to short span, transient and small magma bodies (or mushes).

l.68, you may add that details on the numerical method are also provided in Supplemental (not just in other papers), where it would be good to see the main two equations that are solved, eg. the conservation laws for Darcy fluid and for temperature, so that we can identify the key parameters at play. I think it is also important you mention here (sooner than as it is now) that both permeability, viscosity, (and density ?? what about it?) have prescribed evolution as a function of space and/or temperature /or crystal fraction.

l.75, the depth distribution of melt lenses follows a specific probability density function, defined with a parameter « beta », and which will determine the proportion of melt at a given depth (1-X), correct (fig.2B) ? please clarify the link between parameters and their meaning, as later on in the text you use only beta or X or Z, better to introduce them first clearly.

l.80, you mention a max. permeability, but not a lower one ?
It is not clear to me, about how you make the distinction between the hydrothermal convecting system and the magmatic convecting system : densities and viscosities are drastically different in between both entities : do you prescribe that difference by just fixing k=e-14 above Z_H, and then below, what is its order of values (min, given the function you define) ? Idem for viscosities ; above Z_H, what is its value ? And what about density variations, although it does not need to be explicit in your convection models, it might be useful for the external reader to grasp that ?

Fig.1 : Although i reckon the figure is « dense » already, i have spent time looking for Z(AML), Z(Moho), AML (a lens, named in the caption but not in the Figure), Nu (the Nusselt number, in the figure but not in the caption).

RESULTS
L.101-107, i find that these statements rather relate to data information than modeling results. I suggest to modify the sentence 98-100/ a slight rewording would help decipher between what is strong solid information from data knowledge and what are your related assumptions (input) and results (outputs) here. Actually, this paragraph describes more a justification of your assumption of the emplacement of magma bodies at first, maybe shift it in the setup section ?

l.108, melt migration is « simplified by assigning », maybe reword, by « approximated » with..

l.113, « As an example, » as a matter of fact no, replace by « This leads to »... a total proportion…

l.116 : you describe your numerical tests range. Could you state more clearly, “we tested 6 values of beta (melt emplacement mode), and 3 values of the hydrothermal layer thickness, leading to 18 test cases.”

You should also inform somewhere that your steady state outputs and analysis will be made 30 kyrs after model onset ? How is that justified?

L119, the crystal fraction field follows a law that was determined experimentally by 48, it is an input (not clear when reading at first), not an output.

l.138 : i don't understand from the figures, that some domains result to be hotter than others ? Given the boundary conditions in temperature, it is difficult to decipher differences in between models ? Or do you just aim to describe the slight change in slope of the black solidus line in figures ? Please clarify.

l.146 : why do you judge that z_H=3km leads to « excessive » cooling in that case ? Be more precise and justify.

l.149, reference to fig. 2D (zH=0) with reference to Fig.2E, F (zH>0) ?

l.179-189 : analytical Nu(Ra) relationship, in question. I know that this relationship with Ra is classical, but i was wondering whether parts of your magmatic system might actually fall into more complex regimes which might be worth mentioning, or « contextualise », depending actually on the Prantl number. For instance Gerbault et al., 2025 argued that a slightly more complex relationship would apply at the boundary between partially molten basalt and rhyolite. Has anyone studied that for the partially molten oceanic ridge basaltic systems? according to Silano et al., 2010 : « In the range of Ra and Pr considered, the flow passes through several transitional phases, and a single power law is expected to be inadequate to describe the Nu scaling in different regimes (Grossmann & Lohse 2000). » cf. their Fig.16 Nu(Pr), independent of Pr.

According to Grossman & Lohse 2001 : « … a linear combination of the 1/4 and the 1/3 power laws for Nu with Ra, Nu = 0.27Ra1/4 + 0.038Ra1/3 (prefactors follow from experiment), mimics a 2/7 power-law exponent in a regime as large as ten decades. » Cf. Their Fig.2. By how much would this change your deductions ?
This also brings the question of the numerical mesh resolution of your models that is required to correctly reproduce the heat transfer mode, could you refer to previous works in which you will probably have tested that (for instance in appendix)?

l.181 ; it is unclear here (you may recall) where or how the nature of D is measured : is it controlled as an input with the amount of injected melt lens, or is it the sum of magmatic domain with X>Xc measured at the end of the model time (steady state) ?
Also please precise here that you consider kinematic or dynamic viscosity as nu_m vs. nu_0 is provide in m²/s.

Now what does the evaluation of Nu brings to the better understanding of the magmatic system ? How does it link to a better formulation of the heat transfer mode over time (just an extra sentence).

l.197 : you develop your exploration models by switching on magmatic convection now : could you detail how you do this technically (add a term in solved equations, modify a parameter that enables it)?

l.204 : i suggest in appendix you reproduce one of those figures from your previous papers that allow the reader to better visualise the natural system that is modelled.

l.204, occurring should be « occur ». Verb tense, correct ?

l.206 : here you say you measure Dmax, which recalls my previous question of how D is defined as input/output l.181 (X>Xc in in put or end of model?).

Fig.4 and 5, i would like to see more clearly in the figures, how you define the convecting system/domains in your models ? Is that where temperature is uniform for a while, but then what is the technical « indicator » or threshold?

l.222 « for the same beta », recall here what beta is, as you do in fact l.227 (the initial depth distribution of melt crystallisation, in 1/m),

l.224, would you also say something on how this pronounced transience is independent of the hydrothermal circulation intensity ? (add a sentence). Could you also precise/recall here the characteristic time-scale of this transient phenomenon/ which over /range of time-scales and space scales (1 km, 30ky?)

l.227, i am not sure i get it, that the overall thermal evolution is actually controlled rather by the amount of heat brought into the system, which is not varied, and the definition of permeability and other properties with temperature that actually tend to « fix » the thermal distribution. Maybe you have in mind another more basic standard ancient model in which the thermal distribution might have been more linear and simple ?
I am not sure what you have in mind considering the contribution to cooling by the effect of the overlying hydrothermal system : could you perhaps illustrate this -for instance in appendix- as of the relative contributions typical from each process (X melt distribution, magma convection, and hydrothermal system?). Maybe recall previous work on this.

l.249, i suggest to rephrase this sentence, something like, “To investigate the impact of convection on magma lifespan, we run addition simulations for which we perform a Monte Carlos analysis, on single injection events restarted from the final stage obtained in the previous simulations. »

l.256 : 0.1 km « increments » of what ? Do you mean you place 100 m of melt lenses from 1.5 to 5 km depth ? Every 0.1 km thickness ? Separated by 0.1 km ?

l.267-.. 2.2 km depth, 3.5 km depth, add depth... « and finally stabilizes up to 5 km » : unclear, do you mean when it is finally emplaced at the maximum depth of 5 km ?

l.279, i suggest to rephrase : « melt pockets with a lifespan less than 4 years ».

l.282, split the sentence in two after the parenthesis.. « These results imply... »

l.286 ; applicability of your modelled scenario to continental systems. I think that as written here this is far fetched. First, moderate « are equally », by « may be ». Then you don’t provide any reference supporting this statement. And you should mention the moderate similarity of the crystal to melt relationship in more felsic systems, as well as how that may lead to similar convecting systems but at similar or distinct scales etc.. ; there is a chain of processes and assumptions that deserve discussion prior to jumping to that conclusion that the systems are equivalent. All the more that you make this claims in your conclusion and abstract: you are not demonstrating this by any means here.
Not only do you have lower permeability in continental systems, but melt lenses could be emplaced much deeper therefore diffusive processes might occur much more than what you have here? Viscosity of magmas are not the same, etc etc.. There are a number of differences that i think you should mention prior to claiming this extrapolation. I am not saying that it is not applicable, i am just saying that you may propose this but notifying the naive reader of the difficulties and differences that such an extrapolation implies.
Maybe you could provide references on similar modeling approaches or descriptive studies in ophiolitic systems (Oman) or

in the Afar volcanic area (eg. Ebinger et al., 2010, there might be more suitable ones) ? Your study reminds me of the generic study of Havlin et al. ,2013, considering pulsating magma injections at the base of lithospheres.

l.288 you propose that the highly transient nature of the crystal-poor magma body is a universal behavior on Earth thanks to your modeling results ; i am less enthusiastic than you. Recall that you are assuming already a transient input of 1000 m of heated magma every 200 yrs. Previous studies have allowed you to tune this value, nevertheless, this value remains a strong first order assumption of your modeling approach ! What if you had continuous supply at a lower rate ? What if you had stronger irregular pulses of magma injection ? So what you can best rather deduce from your models, is that, given the assumption of a periodic magma input rate at the base of your modelled domain, you manage to reproduce the development of transient magma reservoir lifespan reduced to 2 % ; meaning 4 years for 200 years of periodical basal injection. That is already a very good result and i think you should stick to that without claiming more than what the model displays.

l.305 :."..may experience multiple episodes of remelting and recrystallisation over several years timespan," etc. : this is a result i would emphasize in the conclusion and abstract of this ms. Instead of the above (applicability to continental settings) on which i don't agree so much.

CONCLUSIONS

l.323, « shortens the lifespan of eruptible magma bodies by 2 orders of magnitudes », i suggest you add here your quantitative result in parenthesis « (in fact here years) »,

l.328 : delete « continental » magma-mush systems, unless you provide more evidence of the applicability of your model to continental systems. Or add, « and to some extent continental as well».

l.331, maybe you want to say here « fossil » magma plumbing systems.. Do you have some in mind like Oman or others (references?eg. Nicolas & Boudier papers ?)

Suggested references:
Ebinger, C., Ayele, A., Keir, D., Rowland, J., Yirgu, G., Wright, T., ... & Hamling, I. (2010). Length and timescales of rift faulting and magma intrusion: The Afar rifting cycle from 2005 to present. Annual Review of Earth and Planetary Sciences, 38(1), 439-466.
Gerbault, M., Melnik, O., & Borisova, A. (2025). Numerical simulation of magma-rock interaction at Krafla volcano using OpenFOAM software and a simplified thermal model. Solid Earth, 16(11), 1307-1333.
Grossmann, S., & Lohse, D. (2001). Thermal convection for large Prandtl numbers. Physical review letters, 86(15), 3316.
Gutiérrez, F., Payacán, I., Gelman, S. E., Bachmann, O., & Parada, M. A. (2013). Late-stage magma flow in a shallow felsic reservoir: Merging the anisotropy of magnetic susceptibility record with numerical simulations in La Gloria Pluton, central Chile. Journal of Geophysical Research: Solid Earth, 118(5), 1984-1998.
Havlin, C., Parmentier, E. M., & Hirth, G. (2013). Dike propagation driven by melt accumulation at the lithosphere–asthenosphere boundary. Earth and Planetary Science Letters, 376, 20-28.
Silano, G., Sreenivasan, K. R., & Verzicco, R. (2010). Numerical simulations of Rayleigh–Bénard convection for Prantl numbers between 10– 1 and 104 and Rayleigh numbers between 105 and 109. Journal of fluid mechanics, 662, 409-446.

(Remarks on code availability)
I have checked the availability of files on the indicated web link, input (code and initial parameters) and output (videos), but i have not reran the models in detail.

Reviewer #3

(Remarks to the Author)
Comments on NCOMMS-25-85673 keyed by line number from pdf sent to review. The Journal should always send out pdf's with page numbers. This helps the reviewer.

Line 40: 'and subduction zone magmatism' is better than subductions!

Line 42: this statement needs qualification. Depending on boundary conditions even a mush can undergo convection. For example, with a constant T BC (which may not be appropriate but is used here as an example) with crystal-mush dominated parameters =2600 kg/m3, =5e-5 K-1,g=10 m/s2, T=10 K, L=1 km, =5e-7 m2/s and =1011 Pa s (mush with circa 40% solid fraction, roughly), the canonical Rayleigh number Ra=ραgΔTL3κη equals~3e5 which exceeds the critical value which is of order 103. So even in this state the mush is subject to convection. Now of course other things come into play specifically the relative velocity of interstitial melt and the 'solid' framework---although this 'solid' framework is itself subject to convection. This is very much unlike the simpler situation say in a hydrothermal system where the framework is truly rigid and the only relevant velocity is the Darcy percolation. In a mush it's a bit different. NOTE SYMBOLS DO NOT TRANSLATE WHEN COPIED AND PASTED FROM WORD the properties can be discerned from the units and i am sure the authors will have no problems.

Line 54: ??? clarify the length scale you are claiming to be relevant

Line 61: yes but to evolve from a melt rich system to a mushy one, the system will necessarily move through the melt-

dominated convective regime. This implies that any models that ignore convection are severely compromised and cannot follow the initial stages if newly input magma is relatively aphyric. Reference on rejeuvenation of mush by hot underplating in Nature Reviews Earth & Environment | Volume 6 | June 2025 | 401–416 or American Journal of Science, Vol. 318, January, 2018, P. 90–140, might be useful.

Line 103: the terminology needs to be defined. what is a low-crystallinity mush? Is a mush defined as a magma with a solid fraction >X where X is 40%? 50%, 60% ?then what is a low crystal content mush? The rheological locking crystallinity varies from 52% for a random packing of equal-sized spheres up to 75 % for a serial size mixture of spheres. And its more complicated if one allows for serial sizes AND serial canonical shapes like prisms, tabular platelets, etc.It looks like from figure 3B the 60% threshold is used.

Line 135:for the hydrothermal part of the system are the fluid properties taken as constants? If not specify how fluid density, thermal conductivity, viscosity etc vary. The T-p regime relevant to this study is around the critical point for pure H2O ( 373 C and 218 bars) .In this region of pT space fluid properties vary enormously (see NBS book by Harr on steam tables and properties_

Line 165: what is the thermal boundary condition applied at the boundary between melt body and the mush? And between mush and overlying hydrothermal convection zone? q or T type BC's.

Line 184: more recent discussion of suspension/mush viscosity in magmas by Lesher et al in Encyclopedia of Volcanoes latest addition.

As in any model of a complex system certain aspects have to be left out . Some concerns here include the clear specification of boundary conditions and the treatment of the hydrothermal system , especially the fluid properties. H2O around its critical point has highly variable density , isobaric specific heat capacity, thermal conductivity, isobaric expansivity and dynamic viscosity, all of which affect Darcy Raleigh numbers and hence Nu numbers.

Some of the references could be updated to include more recent ones and are indicated in my comments.

Overall, i think this is a worthwhile study. Although it is hardly surprising that including convection in transient melt lens grossly affects longevity of melt lens and hence influences longevity, this is the first study that although crudely, does attempt to quantify the timescales. The ideas that underplaying can unlock or rejuvenated a mush is not new but their quantification of the unlocking interesting.

(Remarks on code availability)
i do not review code just like i don't do other peoples laundry! i assume they know how to do this. I would only say that part of developing ANY code requires comparing against benchmark existing studies. This is not always possible to do completely but perhaps for 'end member' cases since no two codes are alike. I ASSUME that the authors have done this which is a common practice of professionals.

Version 1:

Reviewer comments:

Reviewer #1

(Remarks to the Author)
The revised manuscript shows clear and substantial improvement. The authors have addressed most of the major concerns raised in the first round. In particular, the model description is now much clearer, the governing equations are explicitly stated, and the role of convection is better explained. The positioning of the work relative to previous studies is also improved, and earlier overstatements (e.g., regarding universality and continental applicability) have been appropriately moderated.

The addition of sensitivity tests is an important and convincing step that strengthens the robustness of the conclusions.

That said, a few points still need clarification:

1. Eq. (5) assumes constant fluid density. This contradicts with the equation of state used in simulations. What is the effect of the fluid compressibility?
2. The neglect of compositional convection is acknowledged, but its implications should be emphasized more clearly.
3. The model imposes periodic melt injection. Only one supply rate is considered. It will be interesting to see the influence of the supply rate on the melt fraction evolution. See Melnik, O., Utkin, I. S., & Bindeman, I. N. (2021). Magma chamber formation by dike accretion and crustal melting: 2D thermo-compositional model with emphasis on eruptions and implication for zircon records. Journal of Geophysical Research: Solid Earth, 126(12), e2021JB023008. https://doi.org/10.1029/2021JB023008
4. The definition of magma body thickness (via the Xc=0.6 threshold) could use a brief comment on sensitivity, as it directly affects the results.

Overall, the paper is now much stronger and close to being publishable. I recommend minor revision to address the points above.

(Remarks on code availability)
code folder is too big because it includes many videos. data should be separated from the code.

Reviewer #2

(Remarks to the Author)
The authors have appropriately adressed comments by all reviewers by their modifications to the original submission. I find that this manuscript is now much better introduced, detailed and discussed within the current state of the art of knowledge on such magmatic processes. Therefore i recommend it for publication.

(Remarks on code availability)

**Open Access** This Peer Review File is licensed under a Creative Commons Attribution 4.0 International License, which permits use, sharing, adaptation, distribution and reproduction in any medium or format, as long as you give appropriate credit to the original author(s) and the source, provide a link to the Creative Commons license, and indicate if changes were made.
In cases where reviewers are anonymous, credit should be given to 'Anonymous Referee' and the source.
The images or other third party material in this Peer Review File are included in the article's Creative Commons license, unless indicated otherwise in a credit line to the material. If material is not included in the article's Creative Commons license and your intended use is not permitted by statutory regulation or exceeds the permitted use, you will need to obtain permission directly from the copyright holder.
To view a copy of this license, visit https://creativecommons.org/licenses/by/4.0/

Reviewer #1:
Here's a polished version of your review with grammar, wording, and consistency cleaned up, but keeping your structure and arguments.
---
The manuscript presents 2-D numerical simulations of a lower-crustal magma–mush system beneath a fast-spreading ridge. The authors build on an existing thermo-mechanical framework in which sill-like melt lenses are repeatedly emplaced into a gabbroic mush between the axial magma lens (AML) and the Moho. Heat is removed by conduction in the crust, by parameterized convection within melt-rich regions (via a Nusselt-number enhancement of thermal diffusivity), and by Darcy-style hydrothermal circulation in a permeable upper layer. The main quantitative result is that including convective heat transport in the magma lenses reduces the lifetime of "mobilizable" melt from hundreds to thousands of years (conduction-only) to at most a few years, while leaving the long-term thermal structure of the mush largely unchanged.

The physical interpretation of the process is correct, but not novel, and its implementation within the mathematical model requires further clarification and adjustment.
We thank the reviewer for the comments and for the opportunity to clarify the novelty of this study. Although magma convection has been considered in a few studies, these studies often focused on the scale of individual magma bodies and did not address the implications for a lithosphere-scale geodynamic framework, such as the context of seafloor spreading in our study. Besides, the transient evolution of magma-mush systems and the lifespan of eruptible magma remain poorly constrained when magma convection is taken into account. A central contribution of our study is to show how magma convection modifies the thermal evolution, crystallization history, and persistence of melt in such systems. We therefore believe that the novelty of this study lies not only in implementing magma convection itself, but also in quantifying its consequences for transient magma-mush evolution in fast-spreading environments.

As for the clarification of mathematical aspects, we added and described governing equations in Methods section.

---

Novelty

1. The role of convective heat transfer during the emplacement of magma into the crust has been well understood since the classical papers by Huppert and Sparks (1988):
Huppert, H.E., Sparks, R.S.J., 1988. Melting the roof of a chamber containing a hot, turbulently convecting fluid. Journal of Fluid Mechanics 188, 107–131.
[https://doi.org/10.1017/S0022112088000655](https://doi.org/10.1017/S0022112088000655)
Huppert, H.E., Sparks, R.S.J., 1988. The Generation of Granitic Magmas by Intrusion of Basalt into Continental Crust. Journal of Petrology 29, 599–624.
[https://doi.org/10.1093/petrology/29.3.599](https://doi.org/10.1093/petrology/29.3.599)

2. The parameterization of effective heat transfer in convective regions was already implemented in, e.g., Annen et al. (2008), where a temperature-averaging procedure was applied to magma-rich regions.
Annen, C., Pichavant, M., Bachmann, O., Burgisser, A., 2008. Conditions for the growth of a long-lived shallow crustal magma chamber below Mount Pelée volcano (Martinique, Lesser

Antilles Arc). Journal of Geophysical Research: Solid Earth 113. [https://doi.org/10.1029/2007JB005049](https://doi.org/10.1029/2007JB005049)

3. Recent papers by Gerbault, Borisova and Melnik introduced an effective heat capacity based on a Nu–Ra parameterization. In the 2025 paper, the simplified 1-D model was compared with a full-scale 2-D numerical simulation.
Gerbault, M., Melnik, O., Borisova, A., 2025. Numerical simulation of magma-rock interaction at Krafla volcano using OpenFOAM software and a simplified thermal model. Solid Earth 16, 1307–1333. [https://doi.org/10.5194/se-16-1307-2025](https://doi.org/10.5194/se-16-1307-2025)
Borisova, A.Y., Melnik, O.E., Gaborit, N., Bindeman, I.N., Traillou, T., Raffarin, M., Stefánsson, A., Laurent, O., Leisen, M., Llovet, X., de Parseval, P., Proietti, A., Tait, S., 2023. In situ probing of the present-day zircon-bearing magma chamber at Krafla, Northeastern Iceland. Frontiers in Earth Science 11.

4. A relevant model by Liu & Buck (2023) is not discussed in the text.
Liu, Z., Buck, W.R., 2023. The spreading rate dependence of the distribution of axial magma lenses along mid-ocean ridges. Proceedings of the National Academy of Sciences 120, e2214048120.
[https://doi.org/10.1073/pnas.2214048120](https://doi.org/10.1073/pnas.2214048120)
We have added and discussed the references above in the revised manuscript. See response below referring to the model by Gerbault et al., 2025.

The model

The model is not well discussed in the paper, and there is also no complete description in the referenced papers. All governing equations should be clearly specified in the Supplementary Material.
1. It is not clear which equations are solved. Is it only the heat equation, or a full advection–diffusion equation? How are the displacements of the magma and mush treated following sill injection?
We have added a dedicated section in the Methods, describing all governing equations in detail.

Magma and mush cool and crystalize in-situ following the intrusion of each sill, see Methods at Line 466: “Each sill emplacement resets the temperature to a uniform liquidus temperature (TL) of 1250°C, corresponding to a crystal fraction (X) of 0. This melt then cools and crystalizes in-situ, releasing specific and latent heat.”
While we do not account for mass flow of the magma and mush in the lower crust, as discussed in the text, our parameterization of depth-dependent sill emplacement (e.g., via the beta factor) can represent melt migration to shallower levels once new melt is emplaced at a deeper structural level.

2. How is the thermal conductivity parameterized? Only Nu(Ra) is discussed in the text.
We added Equation (6) to clarify the parameterization of Nu in thermal conductivity. Specifically, Nu is used as a multiplying factor applied to thermal conductivity, which is otherwise assumed constant and spatially uniform. Outside the magma body, Nu=1. Inside the magma body, Nu>1.

3. How is the Rayleigh number calculated? It depends on the temperature difference and the characteristic vertical extent of the domain (Ra ~ deltaT D^3). How is deltaT defined? As the sill cools down and the mush heats up, the temperature difference progressively decreases. Is D fixed? What happens when mobile magma batches merge?
Both ΔT and D are measured and updated at every time step, as the temperature field evolves. At Line 212: ΔT is defined between Tc (1150°C) and the magma body's maximum temperature, which progressively decreases following sill injection. D is measured vertically. Technically, we extract a contour corresponding to a crystal fraction of 0.6, and then measure the average vertical extent of the magma body defined by this contour. D corresponds to this average.
In cases where several isolated magma bodies exist, we measure ΔT and D for each individual contour, and calculate Ra numbers specific to each body. When magma batches merge, we treat them as a single connected magma body.

4. Gerbault et al. have shown that the main driver for convection can be compositional rather than purely thermal, because densities of melt and crystals for basalt differ significantly. The compositional Rayleigh number is more than an order of magnitude larger than the thermal one. This dependence should be taken into account.
We agree that density contrasts potentially lead to compositional convection between injected magma and crystals. We expect that it would further decrease the lifespan of crystal-poor magma domains, by further enhancing the vigor of convection. The duration we obtain for the magma stage without considering this additional process should thus be considered a maximum.

We added a sentence at Line 230: "We note that Ra in our model only accounts for thermally induced convection, whereas compositional convection arising from density contrasts between basaltic melts and crystals may generate larger Ra (Gerbault et al., 2025)."

And at Line 327: "We expect that compositional convection between magma and crystals would further decrease the lifespan of crystal-poor magma domains (Gerbault et al., 2025); therefore, the magma lifespan obtained in our model, which does not include this additional process, should be regarded as a maximum estimate."

5. It is not clear why the viscosity depends only on the crystal content. Melt viscosity itself is strongly temperature-dependent.
We agree that melt viscosity is also temperature-dependent. In the revised manuscript, we adopt the reference viscosity of liquid basaltic magma (vm0) at 0.01 $m^2/s$, reduced from 0.1 $m^2/s$ in the previous version, following the estimates and references suggested by Reviewer #3 (Lesher and Spera, 2015; Toussaint and France 2026; Giordano et al., 2008).

Following Toussaint and France 2026, the kinematic melt viscosity can be derived from the thermodynamic model Rhyolite-MELTS, which is the same framework used to derive the temperature-crystal fraction relationship (France et al., 2025). We calibrate it to vm0 (0.01 $m^2/s$) at the liquidus temperature of 1250°C. Melt viscosity varies by less than one order of magnitude over the magma temperatures of 1250-1150°C (see figure below). In contrast, magma viscosity, calculated based on crystal fraction, increases by up to 5 orders of magnitude as magma cools. Therefore, although melt viscosity is temperature-dependent, magma viscosity is dominantly controlled by crystal fraction.

We added this discussion and this figure in the Supplementary (Fig. S13).

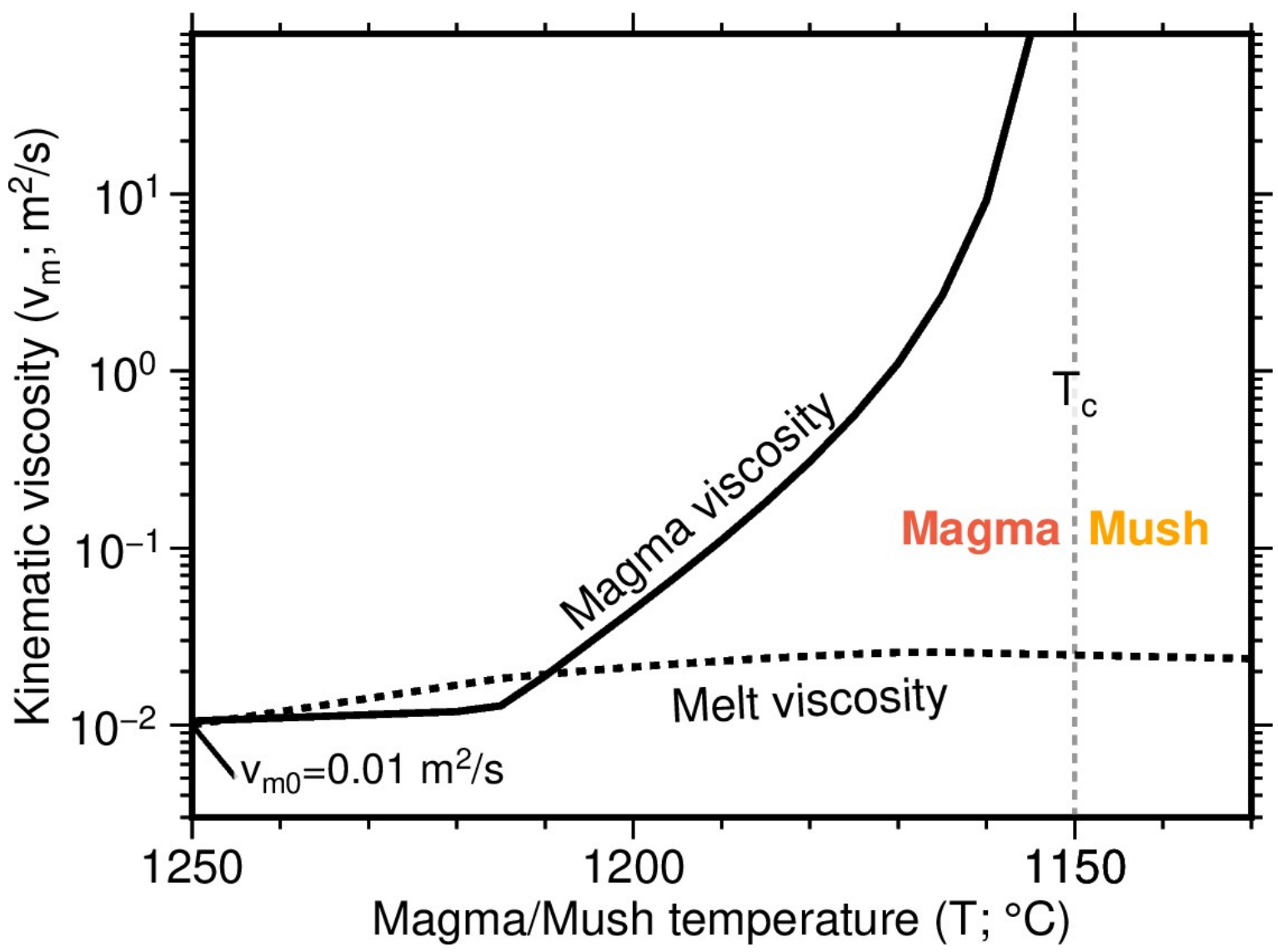


In conclusion, the paper is currently not suitable for publication in Nature Communications. A major revision is required to clarify the model, properly position the work with respect to existing literature, and address the issues listed above.

Reviewer #2

This contribution is a nice build up on previous work from the same group that focusses on the specific question of the lifespan of eruptible magmas below shallow magma bodies, especially below oceanic spreading ridges. The authors consider a shallow 6 km deep magmatic system at the base of which lenses of partially molten material is injected every 200 yrs, and model how coupled hydrothermal assumptions manage to explain how these lenses emplace at ca 1.5 km depth and can last several years to decades, depending on basically 2 assumptions of the initial partial melt distribution within the crustal column and the efficiency (depth) of the overlying hydrothermal system in diffusing temperature. The text is well written and figures and explanations are relatively clear, therefore a majority of my comments below are simple requests to clarify some formal explanations and descriptions.

In order for the reading audience to better grasp the novelty of the approach and the key results, I suggest to slightly reorganise the introduction section so as to make it clearer what is novel view point here with respect to previous studies (most of which by the same group).

We thank the reviewer for their careful assessment of our manuscript and their constructive comments.

We have reorganized the Introduction and added a new section 'Numerical thermal model'. See response below for more details.

Then I have 2 main remarks regarding the conclusions, 1) I find far-fetched to state that those results are applicable to continental settings, while no references nor quantitative arguments are brought here to support the validity of this extrapolation. So either delete that statement or feed in more quantitative demonstration. 2) the conclusion that these models explain the self-consistent pulsating behavior of shallow magmatic systems is also far fetched given that the authors consider periodical magmatic input in their models already (every 200 years) , so self-consistency is not totally demonstrated here, nevertheless they do show neatly which controlling parameters allow to reduce this basal input frequency by 2 orders of magnitude (eg. years).

1) For the application of our model to continental settings, we have rephrased the conclusion: "We therefore propose that magma convection is an inherent process in oceanic magma-mush systems, and, likely in other geodynamic contexts as well."

We also discussed this at Line 337: "Notably, in the absence of hydrothermal circulation (ZH = 0 km), the reduction of magma lifespan due to convection is even more pronounced (Fig. 6B), suggesting that our findings for oceanic spreading centers may be applicable to non-oceanic settings where hydrothermal activity is limited (Ebinger et al., 2010; Gerbault et al., 2025). In particular, our conclusions on the lifespan of melt-rich bodies and their transience can potentially be extended to other mafic systems, with or without hydrothermal systems, where episodic magma recharges through mushy systems are identified. It may be the case at slow spreading oceanic centers (Boulanger et al., 2020; Chen et al., 2021), ocean-continent transitions (Ebinger et al., 2010; Medynski et al., 2015; Pin et al., 2026), or other continental settings (Cashman et al., 2017)."

2) To address the concern regarding the imposed periodic melt input, we performed additional sensitivity tests presented in the Supplementary and Fig. S12. In addition to the reference case (50 m sill thickness, 200-year interval), we tested a thinner sill of 25 m, corresponding to a melt injection interval of 100 years. The results are nearly identical to those of the reference model, suggesting that our conclusions are not controlled by the specific choice of melt injection interval. We have revised the manuscript to avoid overstating self-consistency.

I hope that the authors will be willing to make these little text adjustments in order for the reader to better understand the true added value of this contribution to our understanding of shallow magma systems dynamics. Then obviously, in my opinion it will well deserve publication.

INTRODUCTION

The first two paragraphs are well informative, but an additional sentence would be useful, that somehow would state more explicitly what your previous contributions (at least 4) or those of others have brought in terms of new insight and what you propose here as additional insight, because somehow it is not clear how by using the same numerical method, you are going to look here at a slightly different aspect, or setup or time-scale, that will more specifically answer the question of the lifespan of eruptible magmas below shallow magma bodies. One or two sentences should be sufficient. This goes with the flaws of such publications that we need to go way back to previous authors contributions to understand the progression in developments and gained insight, it is heavy for the reader that does not necessarily follow the subject closely.
Following these helpful suggestions, and in agreement with the suggestions of the other reviewers, we now frame the objectives of the paper more explicitly in the context of prior studies. This is done in the Introduction at Line 67: "At submarine magmatic systems, magma convection has been widely omitted in most thermo-kinetic models (Chen et al., 2023, 2022; Fan et al., 2021; Liu and Buck, 2023; Maclennan et al., 2004; Morgan and Chen, 1993; Theissen-Krah et al., 2016)."

We also added a new section "Numerical thermal model", stating our previous model's functions and highlighting the novel features designed for the present study.

Beginning of second paragraph you broaden the contextual application of shallow magma bodies to spreading ridges, hotspots and volcanic arcs. You may add la Réunion to Hawaii which is not a unique hotspot, and Andean or other volcanic arcs and not just Cascadia. Convection in shallow volcanic arc magma bodies have also been studied in for instance Chilean plutons eg. Gutierrez et al. (2013) I find a bit restrictive to only mention well known features from the Northern America community with respect to other volcano-tectonic settings worldwide.
We have broadened the references for magma plumbing systems such as la Réunion and Andes, as well as the convection studies like Iceland, Chilean plutons, and arc volcanoes (Martinique Island).

Second paragraph again, you may mention in the context of isolated transient and shallow magma bodies, the recent study by Gerbault et al., 2025 who modelled convection and heat transfer within a 2km deep shallow sill below Krafla, over 35 to 300 yrs. This goes with the question raised, l.59-61, of how widespread magma bodies « live » with respect to short span, transient and small magma bodies (or mushes).
We added a sentence for this recent study at Line 65: "At Krafla volcano in particular, magma convection within a shallow (~2 km) melt intrusion can substantially enhance heat transfer to the surrounding cold host rocks over timescales of 35-300 years (Gerbault et al., 2025)".

l.68, you may add that details on the numerical method are also provided in Supplemental (not just in other papers), where it would be good to see the main two equations that are solved, eg. the conservation laws for Darcy fluid and for temperature, so that we can identify the key parameters at play. I think it is also important you mention here (sooner than as it is now) that both permeability, viscosity, (and density ?? what about it?) have prescribed evolution as a function of space and/or temperature /or crystal fraction.
We have added all governing equations (Equations 5 and 6) to the Methods section. Key parameters are also introduced in the section "Numerical thermal model".

l.75, the depth distribution of melt lenses follows a specific probability density function, defined with a parameter « beta », and which will determine the proportion of melt at a given depth (1-X), correct (fig.2B) ? please clarify the link between parameters and their meaning, as later on in the text you use only beta or X or Z, better to introduce them first clearly.
This is correct. We clarified these parameters at Line 106: "The depth distribution of melt sills (or of melt crystallization) is prescribed by a normalized probability density function (PDF) that decays exponentially with depth, where β is the decay rate (in m-1). This parameter determines the time-averaged proportion of melt crystallized at a given depth (Fig. 2A), or within a given depth range (Fig. 2B)."

l.80, you mention a max. permeability, but not a lower one ?
It is not clear to me, about how you make the distinction between the hydrothermal convecting system and the magmatic convecting system : densities and viscosities are drastically different in between both entities : do you prescribe that difference by just fixing k=e-14 above Z_H, and then below, what is its order of values (min, given the function you define) ? Idem for viscosities ; above Z_H, what is its value ? And what about density variations, although it does not need to be explicit in your convection models, it might be useful for the external reader to grasp that ?
We added a few sentences in the Methods for the minimum permeability and the treatment in the impermeable zone at Line 452: "$Z_H$ and $T_H$ together define the boundary between permeable and impermeable lithosphere, e.g. a fracture front, beneath which permeability is assigned a very low value ($k = 10^{-25}$ $m^2$, effectively impermeable) in the numerical model. Fig. S1 shows snapshots of permeability structures that correspond to models in Fig. 2E ($Z_H$ = 1.5 km) and Fig. 2F ($Z_H$ = 3 km). The minimum permeability, located at x = 8 km, reaches $5.5\times10^{-17}$ $m^2$ and $1.5\times10^{-18}$ $m^2$ for the two cases, respectively."

We added the treatments of hydrothermal fluid properties in Methods at Line 421: "The thermodynamic properties of the hydrothermal fluids correspond to those of a 3.2 wt% NaCl-$H_2O$ mixture (Anderko and Pitzer, 1993; Pitzer et al., 1984). The viscosity of the hydrothermal fluid is determined by temperature, approximated as that of pure water (Holzbecher, 1998)."

Fig.1 : Although i reckon the figure is « dense » already, i have spent time looking for Z(AML), Z(Moho), AML (a lens, named in the caption but not in the Figure), Nu (the Nusselt number, in the figure but not in the caption).
We added $Z_{AML}$ and $Z_{Moho}$ in the figure and the Nusselt number in caption.

RESULTS
L.101-107, i find that these statements rather relate to data information than modeling results. I suggest to modify the sentence 98-100/ a slight rewording would help decipher between

what is strong solid information from data knowledge and what are your related assumptions (input) and results (outputs) here. Actually, this paragraph describes more a justification of your assumption of the emplacement of magma bodies at first, maybe shift it in the setup section ?
We agree these statements primarily provide geological context rather than model results. We have moved this part into to Introduction, serving as background information on fast-spreading ridge systems.

l.108, melt migration is « simplified by assigning », maybe reword, by « approximated » with..
Done

l.113, « As an example, » as a matter of fact no, replace by « This leads to »... a total proportion…
Done

l.116 : you describe your numerical tests range. Could you state more clearly, "we tested 6 values of beta (melt emplacement mode), and 3 values of the hydrothermal layer thickness, leading to 18 test cases."
Done

You should also inform somewhere that your steady state outputs and analysis will be made 30 kyrs after model onset ? How is that justified?
We modified the statement to clarify the timing and the reason of the measurement period, at Line 148: "We measure the maximum thickness (Dmax) of the magma body, i.e., the region where X > Xc, every 0.1 year over a 30 kyrs period following the establishment of a quasi-steady thermal regime (e.g., stabilized solidus geometry and hydrothermal circulation). This time window was empirically found sufficient to capture the full range of temporal variations in the magma body."

L119, the crystal fraction field follows a law that was determined experimentally by 48, it is an input (not clear when reading at first), not an output.
Yes, the temperature-crystal fraction relation is prescribed. To improve clarity, we have revised the sentence in 'Numerical thermal model' section at Line 116: "Crystal fraction (X) of magma and mush is prescribed based on constraints from a thermodynamic model and evolves with temperature (T) according to a nonlinear T-X relationship (France et al., 2025)"

l.138 : i don't understand from the figures, that some domains result to be hotter than others ? Given the boundary conditions in temperature, it is difficult to decipher differences in between models ? Or do you just aim to describe the slight change in slope of the black solidus line in figures ? Please clarify.
We clarify that we are not referring to thermal variations across the Moho, but rather to contrasts within the lower gabbroic layer (>2.5 km depth).
We clarified this at Line 164: "For example, at ZH = 1.5 km (Fig. 2E), the thermal regime in the lower gabbroic layer (>2.5 km depth) becomes colder with increasing β, while the upper-gabbroic layer becomes hotter."

l.146 : why do you judge that z_H=3km leads to « excessive » cooling in that case ? Be more precise and justify.
We clarified the statement about ZH=3 km at Line 172: "Simulations with ZH = 3 km exhibit overall colder thermal regimes compared to those with ZH = 1.5 km, due to more efficient heat extraction by a deeper-penetrating hydrothermal system (Fig. 2F). Specifically, at β = 0 m-1 (ZH = 3 km), the mush zone and the magma body become deeper and smaller, and at β = 4 m-1 (ZH = 3 km), an isolated mush zone forms within the upper-gabbroic layer."

l.149, reference to fig. 2D (zH=0) with reference to Fig.2E, F (zH>0) ?
Done.

l.179-189 : analytical Nu(Ra) relationship, in question. I know that this relationship with Ra is classical, but i was wondering whether parts of your magmatic system might actually fall into more complex regimes which might be worth mentioning, or « contextualise », depending actually on the Prantl number. For instance Gerbault et al., 2025 argued that a slightly more complex relationship would apply at the boundary between partially molten basalt and rhyolite. Has anyone studied that for the partially molten oceanic ridge basaltic systems? according to Silano et al., 2010 : « In the range of Ra and Pr considered, the flow passes through several transitional phases, and a single power law is expected to be inadequate to describe the Nu scaling in different regimes (Grossmann & Lohse 2000). » cf. their Fig.16 Nu(Pr), independent of Pr.
According to Grossman & Lohse 2001 : « … a linear combination of the 1/4 and the 1/3 power laws for Nu with Ra, Nu = 0.27Ra1/4 + 0.038Ra1/3 (prefactors follow from experiment), mimics a 2/7 power-law exponent in a regime as large as ten decades. » Cf. Their Fig.2. By how much would this change your deductions ?
This also brings the question of the numerical mesh resolution of your models that is required to correctly reproduce the heat transfer mode, could you refer to previous works in which you will probably have tested that (for instance in appendix)?
We agree that the classic Ra-Nu scaling law is a first-order simplification of heat extraction in an otherwise complex convective system.

To assess the robustness of our results, we conducted two sets of additional sensitivity tests (See more details in Supplementary).
(1) Sensitivity to Nu–Ra scaling: compare models with Nu = $(Ra/Rac)^{1/3}$ (reference model in the main text) and Nu = $(Ra/Rac)^{2/7}$, under the hydrothermal configuration of ZH=1.5 km and beta of 0-4 $m^{-1}$. (Fig. S14). The resulting thermal regimes and magma body transience differ only slightly, and the primary trends and conclusions remain unchanged.

(2) Sensitivity to numerical mesh resolution: We added a comparison of models with grid cell sizes of 50 m (reference model in the main text) and 25 m, under the hydrothermal configuration of ZH=1.5 km and beta of 0-4 $m^{-1}$. (Fig. S12). The differences in thermal regime and magma body transience are also minor, indicating that the adopted mesh resolution adequately captures the heat-transfer behavior in our parameter range.

Two comments below are combined, as they both refer to the measurements of D and Dmax.
l.181 ; it is unclear here (you may recall) where or how the nature of D is measured : is it controlled as an input with the amount of injected melt lens, or is it the sum of magmatic domain with X>Xc measured at the end of the model time (steady state) ?

Also please precise here that you consider kinematic or dynamic viscosity as nu_m vs. nu_0 is provide in m²/s.
l.206 : here you say you measure Dmax, which recalls my previous question of how D is defined as input/output l.181 (X>Xc in in put or end of model?).
In our model, D is not prescribed as an input parameter, but measured (output) over the entire simulation period, and used to calculate Ra for each individual magma body at each timestep.

D is defined as the average thickness of individual domains where X>Xc.
When multiple isolated magma bodies are present, D is measured for each body, and the corresponding Ra and Nu are calculated separately.

We have clarified that we are working with the magma kinematic viscosity ($\nu_m$ in $m^2$/s).

Now what does the evaluation of Nu brings to the better understanding of the magmatic system ? How does it link to a better formulation of the heat transfer mode over time (just an extra sentence).
We added an extra sentence at Line 227: "This demonstrates that a Nusselt-based parameterization of heat extraction dynamically captures the transition from convection-dominated to conduction-dominated heat transfer as magma cools and crystallizes."

l.197 : you develop your exploration models by switching on magmatic convection now : could you detail how you do this technically (add a term in solved equations, modify a parameter that enables it)?
We added details of on how Nu is applied in the model at Line 233 and Equation 6: "We run models incorporating magma convection (Fig. 4 and 5), using Nu to parameterize the enhancement of conductive heat transfer (Nu > 1 within the crystal-poor magma body and Nu = 1 elsewhere), as implemented in Equation (6) (see Methods)."

l.204 : i suggest in appendix you reproduce one of those figures from your previous papers that allow the reader to better visualise the natural system that is modelled.
We are not sure which figure from our previous work the reviewer has in mind, as we do not believe that an equivalent figure was presented there. To improve the visualization, we now refer to Fig. 1A in the revised manuscript, which illustrates the upper gabbro (dark orange) and lower gabbro (light yellow) layers.

l.204, occurring should be « occur ». Verb tense, correct ?
Done.

Fig.4 and 5, i would like to see more clearly in the figures, how you define the convecting system/domains in your models ? Is that where temperature is uniform for a while, but then what is the technical « indicator » or threshold?

The convecting domain is defined as regions where the crystal fraction X < 0.6, corresponding to the magma body in which the Nusselt number Nu > 1 is applied. This criterion is now explicitly stated in the revised figure caption: "Red contour outlines the magma body, defined as regions with crystal fraction (X) < 0.6, where magma convection is parameterized (Nu > 1)."

l.222 « for the same beta », recall here what beta is, as you do in fact l.227 (the initial depth distribution of melt crystallisation, in 1/m),
Done.

l.224, would you also say something on how this pronounced transience is independent of the hydrothermal circulation intensity ? (add a sentence). Could you also precise/recall here the characteristic time-scale of this transient phenomenon/ which over /range of time-scales and space scales (1 km, 30ky?)
We rephrased this sentence at Line 262:" In summary, for a given β (i.e., the depth distribution of melt crystallization, in m-1), simulations incorporating magma convection produce a more transient magma body system than those without convection, with deeper hydrothermal cooling further enhancing the transience. This pronounced transience, such as the maximum thickness of magma body at the simulation with β = 2 m-1 and ZH = 1.5 km varying between 0-0.6 km over 8 kyr (Fig. 4C), is also consistent with petro-structural observations from fossilized oceanic crust."

l.227, i am not sure i get it, that the overall thermal evolution is actually controlled rather by the amount of heat brought into the system, which is not varied, and the definition of permeability and other properties with temperature that actually tend to « fix » the thermal distribution. Maybe you have in mind another more basic standard ancient model in which the thermal distribution might have been more linear and simple ?
I am not sure what you have in mind considering the contribution to cooling by the effect of the overlying hydrothermal system : could you perhaps illustrate this -for instance in appendix- as of the relative contributions typical from each process (X melt distribution, magma convection, and hydrothermal system?). Maybe recall previous work on this.

We added the reference of Fig. S5-S7 and recalled our previous numerical work (Chen et al., 2022@JGR), in which the efficiency of hydrothermal heat extraction can be influenced by the depth of melt emplacement. For the same total heat input, shallower melt emplacement, results into more efficient hydrothermal heat extraction, and a colder thermal regime in surrounding rocks.

See revision at Line 267: "By contrast, the geometry of the mush reservoir is weakly influenced by magma convection (e.g., Fig. 2 and 4), with the axial profiles of temperature and crystal fraction within the mush zone largely similar with and without magma convection (Fig. S5-S7). Instead, it is primarily controlled by the β value and the hydrothermal configuration; a higher β value and a deeper hydrothermal system, i.e., a closer spatial proximity between melt emplacement and the hydrothermal system, lead to more efficient hydrothermal heat extraction (Chen et al., 2022) and, consequently, to a smaller mush zone (Fig. 4D-4F)."

l.249, i suggest to rephrase this sentence, something like, "To investigate the impact of convection on magma lifespan, we run addition simulations for which we perform a Monte Carlos analysis, on single injection events restarted from the final stage obtained in the previous simulations. »
Done.

l.256 : 0.1 km « increments » of what ? Do you mean you place 100 m of melt lenses from 1.5 to 5 km depth ? Every 0.1 km thickness ? Separated by 0.1 km ?
We rephrased this sentence at Line 302: "For each selected temperature field, we run 36 simulations, each with a single injection of a 1000-m-wide and 50-m-thick melt sill emplaced at a depth between 1.5 to 5 km, with 0.1 km increments of depth."

This means that the 36 simulations correspond to a single melt sill emplacement at depths of 1.5, 1.6, …, 4.9, and 5.0 km, respectively.

l.267-.. 2.2 km depth, 3.5 km depth, add depth... « and finally stabilizes up to 5 km » : unclear, do you mean when it is finally emplaced at the maximum depth of 5 km ?
Done. We rephrased this sentence at Line 315: "magma lifespan increases from 180 to 700 years as the depth of melt emplacement increases from 1.5 to 2.2 km depth, then decreases to 100 year at 3.5 km depth, and finally remains relatively constant for emplacement depths between 3.5 and 5 km"

l.279, i suggest to rephrase : « melt pockets with a lifespan less than 4 years ».
Done.

l.282, split the sentence in two after the parenthesis.. « These results imply... »
Done.

l.286 ; applicability of your modelled scenario to continental systems. I think that as written here this is far fetched. First, moderate « are equally », by « may be ». Then you don't provide any reference supporting this statement. And you should mention the moderate similarity of the crystal to melt relationship in more felsic systems, as well as how that may lead to similar convecting systems but at similar or distinct scales etc.. ; there is a chain of processes and assumptions that deserve discussion prior to jumping to that conclusion that the systems are equivalent. All the more that you make this claims in your conclusion and abstract: you are not demonstrating this by any means here.
Not only do you have lower permeability in continental systems, but melt lenses could be emplaced much deeper therefore diffusive processes might occur much more than what you have here? Viscosity of magmas are not the same, etc etc.. There are a number of differences that i think you should mention prior to claiming this extrapolation. I am not saying that it is not applicable, i am just saying that you may propose this but notifying the naive reader of the difficulties and differences that such an extrapolation implies.
Maybe you could provide references on similar modeling approaches or descriptive studies in ophiolitic systems (Oman) or in the Afar volcanic area (eg. Ebinger et al., 2010, there might be more suitable ones) ? Your study reminds me of the generic study of Havlin et al. ,2013, considering pulsating magma injections at the base of lithospheres.
We replaced "are equally" by "may be", added relevant references, and rephrased the sentence at Line 337: "Notably, in the absence of hydrothermal circulation (ZH = 0 km), the reduction of magma lifespan due to convection is even more pronounced (Fig. 6B), suggesting that our findings for oceanic spreading centers may be applicable to non-oceanic settings where hydrothermal activity is limited (Ebinger et al., 2010; Gerbault et al., 2025). In particular, our conclusions on the lifespan of melt-rich bodies and their transience can potentially be extended to other mafic systems, with or without hydrothermal systems, where episodic magma recharges through mushy systems are identified. It may be the case at slow spreading oceanic centers (Boulanger et al., 2020; Chen et al., 2021), ocean-continent

transitions (Ebinger et al., 2010; Medynski et al., 2015; Pin et al., 2026, 2024), or other continental settings (Cashman et al., 2017).”

Overall, we have toned down the implications for continental settings to mainly point out the broader applicability of the physics that we explore here.

l.288 you propose that the highly transient nature of the crystal-poor magma body is a universal behavior on Earth thanks to your modeling results ; i am less enthusiastic than you. Recall that you are assuming already a transient input of 1000 m of heated magma every 200 yrs. Previous studies have allowed you to tune this value, nevertheless, this value remains a strong first order assumption of your modeling approach ! What if you had continuous supply at a lower rate ? What if you had stronger irregular pulses of magma injection ? So what you can best rather deduce from your models, is that, given the assumption of a periodic magma input rate at the base of your modelled domain, you manage to reproduce the development of transient magma reservoir lifespan reduced to 2 % ; meaning 4 years for 200 years of periodical basal injection. That is already a very good result and i think you should stick to that without claiming more than what the model displays.
We deleted this sentence.

l.305 :.”..may experience multiple episodes of remelting and recrystallisation over several years timespan,” etc. : this is a result i would emphasize in the conclusion and abstract of this ms. Instead of the above (applicability to continental settings) on which i don’t agree so much.
Done and emphasized this result in the conclusion and abstract.

CONCLUSIONS

l.323, « shortens the lifespan of eruptible magma bodies by 2 orders of magnitudes », i suggest you add here your quantitative result in parenthesis « (in fact here years) »,
Done.

l.328 : delete « continental » magma-mush systems, unless you provide more evidence of the applicability of your model to continental systems. Or add, « and to some extent continental as well».
Rephrased at Line 385: “We therefore propose that magma convection is an inherent process in oceanic magma-mush systems, and, likely in other geodynamic contexts as well.”

l.331, maybe you want to say here « fossil » magma plumbing systems.. Do you have some in mind like Oman or others (references?eg. Nicolas & Boudier papers ?)
We modified this conclusion at Line 386: “The highly transient nature of magma bodies appears to be a universal characteristic, providing new insights into volcanic eruptibility and suggesting a potential link between geophysical snapshots and the broader architecture of magma plumbing systems across different geodynamic contexts.”

Suggested references:
Ebinger, C., Ayele, A., Keir, D., Rowland, J., Yirgu, G., Wright, T., ... & Hamling, I. (2010). Length and timescales of rift faulting and magma intrusion: The Afar rifting cycle from 2005 to present. Annual Review of Earth and Planetary Sciences, 38(1), 439-466.
Gerbault, M., Melnik, O., & Borisova, A. (2025). Numerical simulation of magma-rock interaction at Krafla volcano using OpenFOAM software and a simplified thermal model. Solid Earth, 16(11), 1307-1333.
Grossmann, S., & Lohse, D. (2001). Thermal convection for large Prandtl numbers. Physical review letters, 86(15), 3316.
Gutiérrez, F., Payacán, I., Gelman, S. E., Bachmann, O., & Parada, M. A. (2013). Late-stage magma flow in a shallow felsic reservoir: Merging the anisotropy of magnetic susceptibility record with numerical simulations in La Gloria Pluton, central Chile. Journal of Geophysical Research: Solid Earth, 118(5), 1984-1998.
Havlin, C., Parmentier, E. M., & Hirth, G. (2013). Dike propagation driven by melt accumulation at the lithosphere–asthenosphere boundary. Earth and Planetary Science Letters, 376, 20-28.
Silano, G., Sreenivasan, K. R., & Verzicco, R. (2010). Numerical simulations of Rayleigh–Bénard convection for Prantl numbers between 10− 1 and 104 and Rayleigh numbers between 105 and 109. Journal of fluid mechanics, 662, 409-446.

Reviewer #2 (Remarks on code availability):

I have checked the availability of files on the indicated web link, input (code and initial parameters) and output (videos), but i have not reran the models in detail.

Reviewer #3
Comments on NCOMMS-25-85673 keyed by line number from pdf sent to review. The Journal should always send out pdf's with page numbers. This helps the reviewer.

Line 40: 'and subduction zone magmatism' is better than subductions!
Done

Line 42: this statement needs qualification. Depending on boundary conditions even a mush can undergo convection. For example, with a constant T BC (which may not be appropriate but is used here as an example) with crystal-mush dominated parameters ρ=2600 kg/m3, α=5e-5 K-1,g=10 m/s2, ΔT=10 K, L=1 km, κ=5e-7 m2/s and η=1011 Pa s (mush with circa 40% solid fraction, roughly), the canonical Rayleigh number Ra=ραgΔTL3κη equals~3e5 which exceeds the critical value which is of order 103. So even in this state the mush is subject to convection. Now of course other things come into play specifically the relative velocity of interstitial melt and the 'solid' framework---although this 'solid' framework is itself subject to convection. This is very much unlike the simpler situation say in a hydrothermal system where the framework is truly rigid and the only relevant velocity is the Darcy percolation. In a mush it's a bit different. NOTE SYMBOLS DO NOT TRANSLATE WHEN COPIED AND PASTED FROM WORD the properties can be discerned from the units and i am sure the authors will have no problems.
We are grateful to the reviewer for the thoughtful and constructive comments.
We have rephased this sentence at Line 45: "Convection can efficiently extract heat when the igneous medium is crystal-poor (i.e., magma), whereas in crystal-rich mushes approaching rheological locking, convective heat transfer is strongly suppressed and cooling primarily occurs by conduction."

Line 54: ??? clarify the length scale you are claiming to be relevant
We clarified the length scale at Line 59: "magma plumbing systems that commonly extend over lateral scales exceeding ~10 km."

Line 61: yes but to evolve from a melt rich system to a mushy one, the system will necessarily move through the melt-dominated convective regime. This implies that any models that ignore convection are severely compromised and cannot follow the initial stages if newly input magma is relatively aphyric. Reference on rejeuvenation of mush by hot underplating in Nature Reviews Earth & Environment | Volume 6 | June 2025 | 401–416 or American Journal of Science, Vol. 318, January, 2018, P. 90–140, might be useful.
We rephrased this sentence to clarify that at Line 72: "Consequently, the broader role of magma convection within mush reservoirs in the thermal regime, crystallization, and the lifespan of eruptible magma bodies remains an open question that warrants further exploration. This issue is especially relevant to the paradigm shift from large crystal-poor magma bodies to widespread crystal-rich mush reservoirs (Bachmann, 2002; Cashman et al., 2017; Humphreys et al., 2025; Sinton and Detrick, 1992), as well as the recognition that newly-injected, nearly crystal-free melts likely evolve through a magma-dominated convective stage (Gerbault et al., 2025; Spera and Bohrson, 2018)."

Line 103: the terminology needs to be defined. what is a low-crystallinity mush? Is a mush defined as a magma with a solid fraction >X where X is 40%? 50%, 60% ?then what is a low crystal content mush? The rheological locking crystallinity varies from 52% for a random packing of equal-sized spheres up to 75 % for a serial size mixture of spheres. And its more complicated if one allows for serial sizes AND serial canonical shapes like prisms, tabular platelets, etc.It looks like from figure 3B the 60% threshold is used.
We do define a clear threshold in Figure 3A. It is at 60% of crystals. This sentence is rephrased at Line 89: "At the EPR 9°50'N, the upper-gabbroic layer likely corresponds to a greatly reduced seismic P-wave velocity (>2 km/s) (Dunn, 2022), potentially comprising multiple seismically-imaged melt sills (Han et al., 2014; Marjanović et al., 2014). In contrast, the lower-gabbroic layer (>2.5 km depth) corresponds to a slightly reduced P-wave velocity (<1 km/s) and very few melt sills (Dunn, 2022; Han et al., 2014; Marjanović et al., 2014, 2023)."

Two comments below are combined, as they all refer to hydrothermal fluid properties.
Line 135:for the hydrothermal part of the system are the fluid properties taken as constants? If not specify how fluid density, thermal conductivity, viscosity etc vary. The T-p regime relevant to this study is around the critical point for pure $H_2O$ ( 373 C and 218 bars) .In this region of pT space fluid properties vary enormously (see NBS book by Harr on steam tables and properties_
As in any model of a complex system certain aspects have to be left out . Some concerns here include the clear specification of boundary conditions and the treatment of the hydrothermal system , especially the fluid properties. $H_2O$ around its critical point has highly variable density , isobaric specific heat capacity, thermal conductivity, isobaric expansivity and dynamic viscosity, all of which affect Darcy Raleigh numbers and hence Nu numbers.

We added governing Equations 5 and 6, which describe thermodynamic properties of the hydrothermal fluids (density, heat capacity, and viscosity). They are not constant but evolve with temperature and pressure conditions in the model, corresponding to those of a 3.2 wt% NaCl-$H_2O$ mixture (Anderko and Pitzer, 1993; Pitzer et al., 1984). The viscosity of the hydrothermal fluid is approximated as that of pure water (Holzbecher, 1998). The thermal conductivity is uniform at 3 W/m/°C.

We agree that fluid properties vary strongly near the critical point. In our model with the maximum hydrothermal temperature at 500°C, hydrothermal fluids may locally exceed the critical temperature, implying supercritical conditions. However, the adopted thermodynamic parameterizations remain valid within this regime and allow continuous variation of fluid properties across the near-critical domain, and capture the first-order heat transport by porous convection in a coupled magma-hydrothermal system.

Line 165: what is the thermal boundary condition applied at the boundary between melt body and the mush? And between mush and overlying hydrothermal convection zone? q or T type BC's.
In our model, the magma body, mush zone, and hydrothermal layer are part of a single continuous computational domain. Therefore, no explicit q or T type boundary conditions are imposed at the interfaces between these regions. Instead, temperature and heat flux are solved continuously across these regions. In melt-rich regions, the Nu-parameterization self-consistently leads to a greatly enhanced thermal conductivity. In regions with any non-zero melt fraction, latent heat if self-consistently included in the heat equation. Lastly, the entire domain is capable of Darcyan convection, but permeability is set to an effectively

impermeable values at depths exceeding ZH and temperatures exceeding TH, which shuts off hydrothermal heat transfers in high-temperature / magmatic regions.

Line 184: more recent discussion of suspension/mush viscosity in magmas by Lesher et al in Encyclopedia of Volcanoes latest addition.
We thank the reviewer for this reference. Following this suggestion, and also considering the comments from Reviewer #1, we further discuss temperature-dependent melt viscosity (see Supplementary and Fig. S12).

In the revised manuscript, we adopt the reference viscosity of liquid basaltic magma (vm0) at 0.01 $m^2/s$ (reduced from 0.1 $m^2/s$ in the previous version). All simulations have been updated. The resulting differences are minor and do not alter the model behavior or the primary conclusions.

Some of the references could be updated to include more recent ones and are indicated in my comments.
Done

Overall, i think this is a worthwhile study. Although it is hardly surprising that including convection in transient melt lens grossly affects longevity of melt lens and hence influences longevity, this is the first study that although crudely, does attempt to quantify the timescales. The ideas that underplaying can unlock or rejuvenated a mush is not new but their quantification of the unlocking interesting.

Reviewer #3 (Remarks on code availability):

i do not review code just like i don't do other peoples laundry! i assume they know how to do this. I would only say that part of developing ANY code requires comparing against benchmark existing studies. This is not always possible to do completely but perhaps for 'end member' cases since no two codes are alike. I ASSUME that the authors have done this which is a common practice of professionals.
As detailed above, we have added a benchmark to test the dependence of our results on grid size. Previous versions of the code have been benchmarked against classical thermal models of mid-ocean ridges (Chen et al., 2023) and analytical solutions (Olive & Crone 2018).

Reviewer #1:

The revised manuscript shows clear and substantial improvement. The authors have addressed most of the major concerns raised in the first round. In particular, the model description is now much clearer, the governing equations are explicitly stated, and the role of convection is better explained. The positioning of the work relative to previous studies is also improved, and earlier overstatements (e.g., regarding universality and continental applicability) have been appropriately moderated.

The addition of sensitivity tests is an important and convincing step that strengthens the robustness of the conclusions.
That said, a few points still need clarification:

1. Eq. (5) assumes constant fluid density. This contradicts with the equation of state used in simulations. What is the effect of the fluid compressibility?

Fluid density is not assumed to be constant in the model. Instead, it depends on temperature and pressure, based on tabulated data (Anderko & Pitzer, 1993; Pitzer et al., 1984). We have clarified this at Line 420: "The thermodynamic properties of the hydrothermal fluids, including ρf, correspond to those of a 3.2 wt% NaCl-H2O mixture [75,76], evolving with temperature and pressure fields in the simulation."

2. The neglect of compositional convection is acknowledged, but its implications should be emphasized more clearly.
We now further emphasize the implications of neglecting compositional convection at Line 324: "We expect that compositional convection between magma and crystals would further decrease the lifespan of crystal-poor magma domains [29]; therefore, the magma lifespan obtained in our model, which does not include this additional process, should be regarded as a maximum estimate. In other words, the transience of eruptible magma bodies in nature may be even greater than estimated in our model.

3. The model imposes periodic melt injection. Only one supply rate is considered. It will be interesting to see the influence of the supply rate on the melt fraction evolution. See Melnik, O., Utkin, I. S., & Bindeman, I. N. (2021). Magma chamber formation by dike accretion and crustal melting: 2D thermo-compositional model with emphasis on eruptions and implication for zircon records. Journal of Geophysical Research: Solid Earth, 126(12), e2021JB023008. https://doi.org/10.1029/2021JB023008

We agree that melt supply rate (i.e., melt flux) is an important control on melt-fraction evolution. In this study, we adopted a single supply-rate formulation (EPR 9N) to isolate the effect of magma convection.
Recognizing that variations in melt supply may also influence melt-fraction evolution, we are currently applying this framework to the Galápagos Spreading Center, where melt flux systematically decreases by ~40% along axis. Decreasing melt flux leads to the formation of increasingly smaller and more transient magma bodies within the mush zone. Preliminary results from this follow-up study were recently presented at EGU: https://meetingorganizer.copernicus.org/EGU26/EGU26-3929.html. We therefore prefer to reserve this important aspect for a dedicated follow-up publication.

4. The definition of magma body thickness (via the Xc=0.6 threshold) could use a brief comment on sensitivity, as it directly affects the results.

We performed sensitivity tests on the critical crystal fraction ($X_c$), comparing values of 0.55, 0.6 (used in the main text), and 0.65. These are now shown in Fig. S15, and commented upon in the supplementary information. For a given $\beta$, all three diagnostic metrics ($D_{max}$, area, and melt volume) increase with increasing $X_c$. The differences are substantial at low $\beta$, but become negligible for $\beta \geq 2\ m^{-1}$.

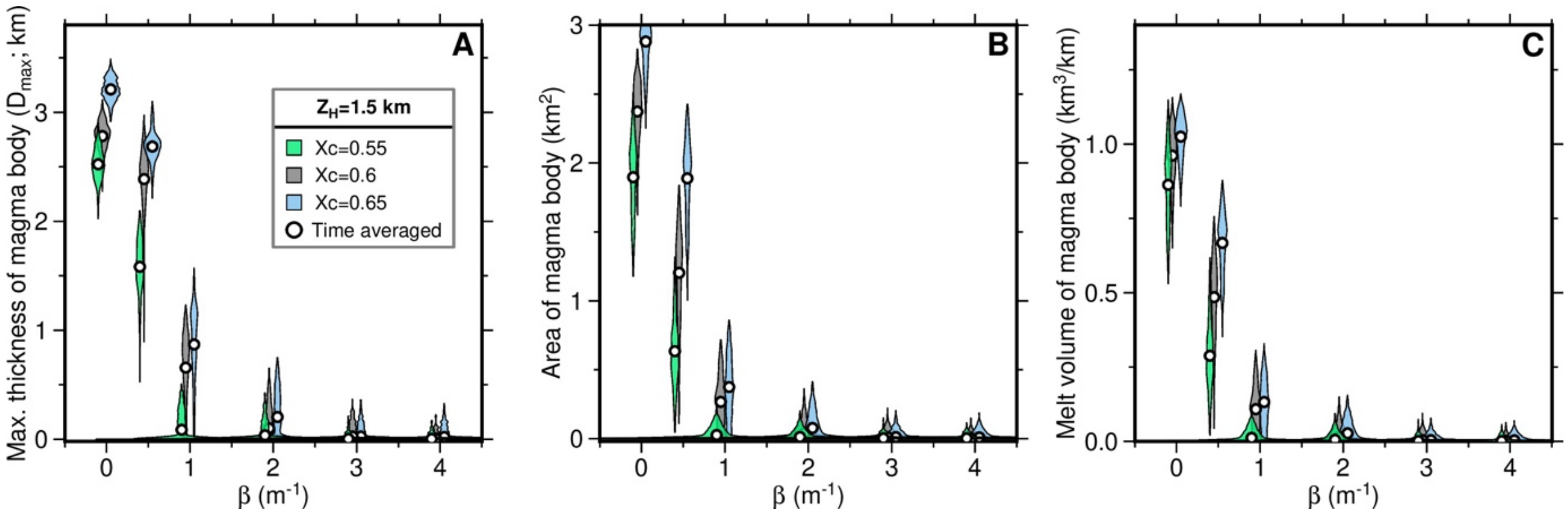


Overall, the paper is now much stronger and close to being publishable. I recommend minor revision to address the points above.

Reviewer #1 (Remarks on code availability):

code folder is too big because it includes many videos. data should be separated from the code.

We made the requested change at https://doi.org/10.6084/m9.figshare.30442703

Reviewer #2:

The authors have appropriately adressed comments by all reviewers by their modifications to the original submission. I find that this manuscript is now much better introduced, detailed and discussed within the current state of the art of knowledge on such magmatic processes. Therefore i recommend it for publication.
Thank you for your positive assessment of our revised manuscript.
